# Inconsistent Patterns of Microbial Diversity and Composition Between Highly Similar Sequencing Protocols: A Case Study With Reef-Building Corals

**DOI:** 10.3389/fmicb.2021.740932

**Published:** 2021-11-25

**Authors:** Hannah E. Epstein, Alejandra Hernandez-Agreda, Samuel Starko, Julia K. Baum, Rebecca Vega Thurber

**Affiliations:** ^1^Department of Microbiology, Oregon State University, Corvallis, OR, United States; ^2^California Academy of Sciences, San Francisco, CA, United States; ^3^Department of Biology, University of Victoria, Victoria, BC, Canada

**Keywords:** amplicon sequencing, 16S rRNA, protocol comparison, coral microbiome, microbial diversity

## Abstract

16S rRNA gene profiling (amplicon sequencing) is a popular technique for understanding host-associated and environmental microbial communities. Most protocols for sequencing amplicon libraries follow a standardized pipeline that can differ slightly depending on laboratory facility and user. Given that the same variable region of the 16S gene is targeted, it is generally accepted that sequencing output from differing protocols are comparable and this assumption underlies our ability to identify universal patterns in microbial dynamics through meta-analyses. However, discrepant results from a combined 16S rRNA gene dataset prepared by two labs whose protocols differed only in DNA polymerase and sequencing platform led us to scrutinize the outputs and challenge the idea of confidently combining them for standard microbiome analysis. Using technical replicates of reef-building coral samples from two species, *Montipora aequituberculata* and *Porites lobata*, we evaluated the consistency of alpha and beta diversity metrics between data resulting from these highly similar protocols. While we found minimal variation in alpha diversity between platform, significant differences were revealed with most beta diversity metrics, dependent on host species. These inconsistencies persisted following removal of low abundance taxa and when comparing across higher taxonomic levels, suggesting that bacterial community differences associated with sequencing protocol are likely to be context dependent and difficult to correct without extensive validation work. The results of this study encourage caution in the statistical comparison and interpretation of studies that combine rRNA gene sequence data from distinct protocols and point to a need for further work identifying mechanistic causes of these observed differences.

## Introduction

Microbial ecology has benefited tremendously from recent technological advances in areas such as high throughput sequencing (Schuster, 2008). The generation of large volumes of genomic data (e.g., 16S rRNA gene sequencing data) has encouraged large-scale collaborative efforts, including the Human Microbiome Project [HMP^1^; (Human Microbiome Project Consortium, 2012)], the Earth Microbiome Project [EMP^2^; (Thompson et al., 2017)] and TARA Oceans (Sunagawa et al., 2015), which aim to catalog all microbial life associated with humans, other animal hosts and across ecosystems. Publicly available sequencing data resulting from these initiatives provide opportunities for the production of meta-analyses and for researchers with smaller scale projects to make comparisons and/or combine their dataset with a much broader set of samples, allowing increased impact of their finer sequencing efforts.

Large-scale collaborations also provide standardized protocols for replication and adequate comparison. For example, the EMP standardized protocols for 16S rRNA gene sequencing are optimized for repeatedly processing large numbers of samples and benefit from automation and high throughput sequencing on an Illumina HiSeq platform. Smaller, individual research laboratories are, in many cases, processing fewer samples less frequently, likely without access to automation, but with the capacity to shift reagents and polymerase chain reaction (PCR) conditions to achieve optimized results. Smaller numbers of samples are also more often sequenced on the Illumina MiSeq platform due to cost effectiveness and increased read length. It has been previously accepted that HiSeq and MiSeq platforms produce comparable results (see Caporaso et al., 2012). In fact, there are few differences in the two sequencing platforms: apart from the discrepancy in read length (HiSeq: 150bp; MiSeq: up to 300bp) and sequencing depth (HiSeq: 150M reads/lane; MiSeq: 20–25M reads/lane), the chemistry between the two methods is almost identical, except for the slightly different concentrations of sodium hydroxide (NaOH) used to denature the libraries for sequencing [HiSeq: 0.1N NaOH; MiSeq: 0.2N NaOH; outlined in (Wu et al., 2018)]. As a result, meta-analyses of 16S rRNA gene data across microbial study systems already utilize cross-protocol and platform data that are stored in public repositories (see Duvallet et al., 2017; Pammi et al., 2017; Mo et al., 2020).

However, when attempting to combine 16S rRNA gene data for a large, longitudinal coral microbiome dataset, we found that the data derived from our in-house preparation and MiSeq sequencing runs clustered separately from those prepared and sequenced by EMP, despite following a highly similar preparation protocol. This led us to re-evaluate if the two protocols utilizing different sequencing platforms provide comparable results. Using 24 coral samples that were sequenced in parallel both in-house (MiSeq) and by EMP (HiSeq), we examined if methodological biases lie within these complex microbial communities, and how (or whether) results obtained from the two protocols are comparable when running standard microbial ecology analyses on alpha diversity, beta diversity, dispersion and differential abundances. Large collaborative sequencing efforts and public sharing of these data are central to understanding general, cosmopolitan patterns in the coral microbiome, which makes effective comparison of sequencing data originating from multiple laboratories vital.

## Materials and Methods

### Sample Collection, DNA Extraction, Library Preparation and Sequencing

Coral samples were originally collected from Kiritimati (Christmas) Island in May 2015 from two species: *Porites lobata* (*n* = 13) and *Montipora aequituberculata* (*n* = 11). Frozen tissue for each individual sample was split in two: one portion was sent directly to EMP (University of California, San Diego) for DNA extraction, PCR, library preparation and sequencing on an Illumina HiSeq 2 × 150bp run (Ul-Hasan et al., 2019) and the other processed in-house at Oregon State University (see previously published methods in McDevitt-Irwin et al., 2019) using a highly similar protocol as EMP but sequenced on an Illumina MiSeq 2 × 300bp run. Both protocols targeted the V4 region of the 16S rRNA gene with the following primers: 515F (Parada et al., 2016) 5′–TCGTCGGCAGCGTCAGATGT GTATAAGAGACAGGTGYCAGCMGCCGCGGTAA–3′ and 80 6R (Apprill et al., 2015) 5′–GTCTCGTGGGCTCGGAGATGTG TATAAGAGACAGGGACTACNVGGGTWTCTAAT– 3′, with the Illumina adapter overhangs underlined. The only difference between the two protocols was the Taq used for PCR: EMP used Platinum Hot Start PCR MasterMix (Thermofisher) and in-house used Accustart^TM^ II PCR ToughMix (QuantaBio). Hereafter, the two protocols will be referred to by their most significant difference: the “HiSeq protocol” run by EMP and the “MiSeq protocol” run in-house.

### Bioinformatics

Sequences from both HiSeq and MiSeq protocol outputs were processed using the QIIME2 pipeline to undergo trimming, quality control, identification of amplicon sequence variants (ASVs), and taxonomic assignment. To ensure comparability between the two protocols and accuracy in ASV-picking, we chose to follow similar treatment of sequencing data by EMP (Thompson et al., 2017); only forward reads were used and trimmed to 120bp. Primers were removed using the plug-in cutadapt (Martin, 2011), and denoising and ASV picking was performed using the DADA2 plug-in (Callahan et al., 2016) on sequences from HiSeq and MiSeq protocols separately, after which were combined into a single dataset for downstream processing. For comparison, ASVs were simultaneously clustered using the plug-in vsearch (Rognes et al., 2016) by 97% similarity, resulting in two output biom tables: one for ASVs and one for 97% clustered operational taxonomic units (hereafter, “OTUs”). Taxonomic assignment for both tables was performed using a naïve Bayes classifier with the SILVA v. 132 database (Quast et al., 2013), trained on each set of representative sequences from the two pipelines.

### Data Import Into R

All statistical analyses were performed in R v. 4.0.2 (R Core Team, 2020); graphics were conducted in R using the package ggplot2 (Wickham, 2016). QIIME feature tables, taxonomic assignments, and tree files for the ASV and OTU datasets were imported into phyloseq (McMurdie and Holmes, 2013) via qiime2R (Bisanz, 2020) for downstream analyses. The SILVA annotations characterized some reads as Phylum: Alphaproteobacteria, Family: Mitochondria. This annotated family contained a mix of bacterial and mitochondrial (eukaryotic) reads: thus eukaryotic mitochondria were further identified using BLASTn (Altschul et al., 1990) and subsequently removed from the two datasets. In the absence of blank controls from the EMP dataset, contaminants were identified using the 4 blank control samples from the MiSeq Data (McDevitt-Irwin et al., 2019). Contaminants were identified and removed (*n* = 102) by prevalence using the decontam package (Callahan, 2020) with a threshold value of 0.5 to ensure all sequences that were more prevalent in negative controls than positive samples were removed. Samples with less than 1000 and 998 reads, respectively, were removed from all analyses for ASV and OTU data. These two numbers differ slightly due to differences in contaminant and mitochondrial read removals as a result of ASV identification versus OTU picking.

### Diversity Metrics and Differential Abundance

We conducted all diversity tests on the two coral species separately due to well-established differences in both alpha and beta diversity measures across host species (Hernandez-Agreda et al., 2017; Epstein et al., 2019; Ziegler et al., 2019) that could have obscured significant differences between protocols. Three alpha diversity metrics were calculated to account for richness, evenness and phylogenetic diversity: observed species richness, Shannon diversity index and Faith’s Phylogenetic Diversity (PD) were calculated on rarefied data (1000 and 998 reads/sample for ASV and OTU data, respectively; these depths were chosen for each dataset to maintain comparability using the highest sample sizes without severely compromising rarefied alpha diversity). For each host species and data type (ASV and OTU), the three alpha diversity metrics were checked for normality using standardized residual plots, Q-Q plots and Shapiro-Wilk tests. If required, log and square-root transformations were performed to meet normality assumptions when data were non-normal (see Supplementary Table 2). Differences in alpha diversity indices between protocols were tested using paired *t*-tests. We also quantified four metrics of beta diversity to examine differences in microbial communities accounting for microbial abundance (Bray-Curtis), presence/absence (binary Jaccard), phylogeny coupled with abundance (weighted UniFrac) and phylogeny coupled with presence/absence (unweighted UniFrac). For each host species, we constructed Bray-Curtis and weighted Unifrac dissimilarity matrices using the relative abundances of taxa to account for differences in sequencing depth between data derived from HiSeq and MiSeq protocols and constructed binary Jaccard and unweighted Unifrac dissimilarity using unrarefied counts. Dissimilarity matrices for all metrics were also built with unrarefied data after removing rare taxa (abundance below 0.5% and 1% threshold per sample). Differences in beta diversity [i.e., both multivariate location (“turnover” and variation)] were tested using permutational analyses of variance (PERMANOVAs) with 999 permutations blocked by coral colony ID (strata = sample label) using the adonis function from the package vegan (Oksanen et al., 2019) implemented in phyloseq (McMurdie and Holmes, 2013). Homogeneity of variances were further tested between protocols using betadisper (PERMDISP) with 999 permutations and communities were visualized using non-metric multidimensional scaling (NMDS) plots. To identify specific significant differences in taxon abundance in the two protocols, differential abundance analyses were performed using DESeq2 (Love et al., 2020) on unrarefied count data with an alpha cut-off of 0.05. All analyses were performed on both ASV and OTU datasets unless otherwise specified. To assess any differences in secondary structure between specific ASVs differentially abundant in HiSeq vs. MiSeq protocols resulting from library denaturation, GC content (%) and melting temperatures were verified through the TmCalculator package in R (Li, 2020). Differences in mean GC content and melting temperature were tested among ASVs present in MiSeq, HiSeq and both protocols using analyses of variance (ANOVAs). We chose to present results from unrarefied data unless otherwise specified in the above methods; however, all analyses were run on both rarefied and unrarefied data and showed no major differences in significance (see Supplementary Tables 3, 5–7).

## Results

### Sequencing Results

To test whether coral microbiome sequence data generated from the two protocols were comparable, we analyzed paired sequence libraries, combined for comparative downstream analyses, using four standard variables for assessing microbiome variations at both the ASV and OTU levels: alpha diversity, beta diversity, beta dispersion, and differential abundance measures. The final ASV dataset included all 24 samples (total *n* = 48 to account for 2 technical replicates per sample, one from each protocol) with a combined total of 1,444,493 reads consisting of 5,512 distinct ASVs for analysis. In the OTU dataset, two *P. lobata* HiSeq protocol samples contained less than 998 reads and were removed along with their MiSeq protocol counterparts leaving 22 samples (total *n* = 44 to account for 2 technical replicates per sample, one from each protocol) for comparison consisting of 953,396 reads and 2,174 OTUs. All comparisons were done using identical read values for both protocols (for sequence read count variation by host species and protocol, see Supplementary Table 1).

### Sensitivity of Alpha Diversity to Protocol

**FIGURE 1:**
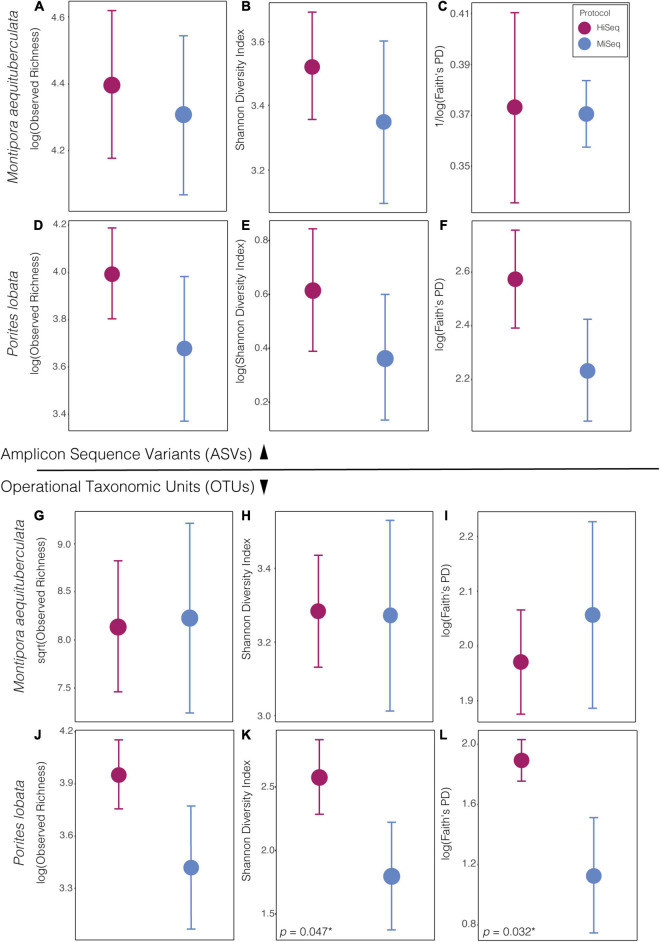
Alpha diversity metrics of bacterial ASVs (top) and OTUs (bottom) between protocols in both species, *Montipora aequituberculata* **(A–C** and **G–I)** and *Porites lobata* **(D–F** and **J–L)**, for observed species richness **(A,D** and **G,F)**, Shannon diversity **(B,E** and **H,K)**, and Faith’s phylogenetic distance **(C,F** and **I,L)** [significant p-values are reported in lower left-hand corner of panel with an asterisk (*). For all other p-values, see Supplementary Table 2].

When using ASVs for analysis, there was a slight tendency for alpha diversity to be lower when calculated from MiSeq protocol data, but diversity did not differ significantly between protocols for any of the four alpha diversity metrics measured (*p* > 0.05: Supplementary Table 2), in either species (Figure 1). However, when using OTUs there was a host species-specific effect on some measures of diversity. Specifically, alpha diversity was not significantly different between protocols for *M. aequituberculata*, but there was protocol sensitivity for *P. lobata* when using Shannon diversity and Faith’s PD measures (but not observed richness), both of which were significantly greater in data resulting from the HiSeq protocol (Figure 1 and Supplementary Table 2).

### Protocol Explains Large Amount of Variation in Community Beta-Diversity

**FIGURE 2:**
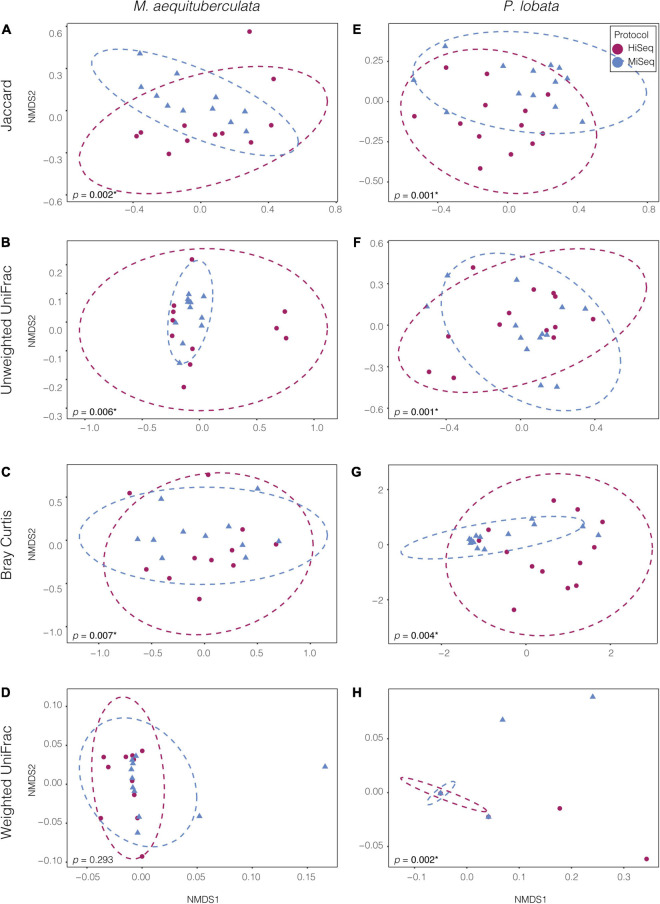
Non-metric multidimensional scaling (NMDS) ordinations of ASV bacterial communities between the two species, *M. aequituberculata* **(A–D)** and *P. lobata* **(E–H)** for each of the four tested dissimilarity metrics: Jaccard, Unweighted UniFrac, Bray-Curtis and Weighted UniFrac. P-values with asterisks (*) refer to significant PERMANOVA results (see Supplementary Table 3).

**FIGURE 3:**
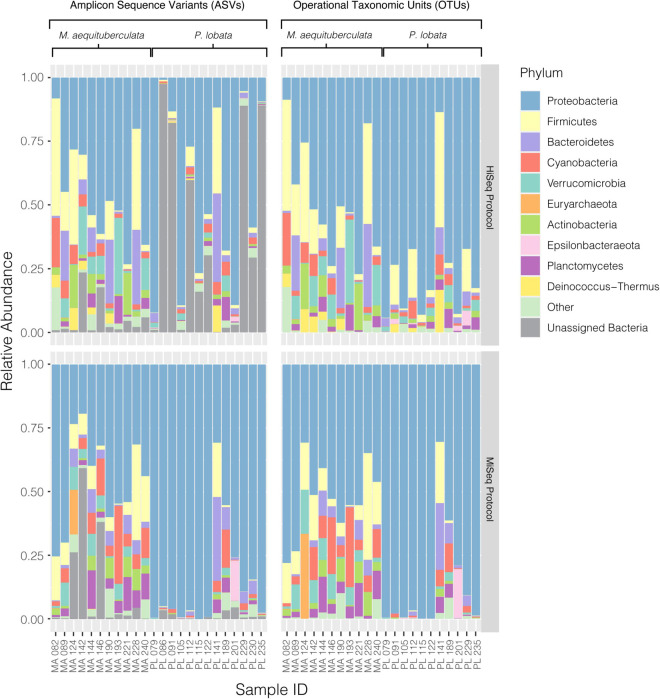
Relative abundances of the top ten most abundant bacterial phyla present in each coral sample from both *M. aequituberculata* and *P. lobata* prepared and sequenced using the HiSeq protocol (top) and the MiSeq protocol (bottom), using both the ASV (left) and OTU (right) datasets. “Other” groups phyla that are not in the top ten most abundant, and “Unassigned Bacteria” refers to unassigned bacterial reads.

Significant differences in microbial community composition were found between protocols for both *M. aequituberculata* and *P. lobata* in all beta diversity metrics except for weighted UniFrac distances for *M. aequituberculata* in both ASV and OTU datasets (Figure 2, Supplementary Figure 1, and Supplementary Table 3). All beta diversity metrics maintained similar dispersions (homogeneity of variances as calculated by the function “betadisper” and referred to as “PERMDISP”; Supplementary Table 3), aside from Bray-Curtis for *P. lobata* in both ASV and OTU datasets (Figure 2G, Supplementary Figure 1G, and Supplementary Table 3), as well as Unweighted UniFrac distances for *M. aequituburculata* in the ASV dataset (Figure 1B and Supplementary Table 3) and *P. lobata* in the OTU dataset (Supplementary Figure 1F and Supplementary Table 3). While the communities did not show consistent, distinct visual segregation of nMDS data clouds according to protocol (Figure 2 and Supplementary Figure 1), some individual samples had highly different relative abundances of bacterial taxa (Figure 3) and community structure (Supplementary Figures 2, 3). While the top 10 most abundant taxa were similar between protocols and across datasets, differences in the relative abundances and detection of some phyla were present (Figure 3 and Supplementary Table 4). In the ASV dataset, seven out of ten phyla were detected in both MiSeq and HiSeq protocols, and phylum-level bacterial community compositions across samples were dominated by Proteobacteria, followed by Firmicutes. However, when clustered as ASVs, these two phyla account for 74.71% versus 54.67% of the composition in MiSeq and HiSeq protocols, respectively, and three different phyla were alternatively detected between the platforms. One of them was phyla Euryarchaeota, which was present in the ASV dataset for MiSeq protocol samples with a mean relative abundance of 4.45% (Supplementary Table 4), but absent in the top 10 most abundant taxa for HiSeq protocol samples, in which the mean relative abundance was less than 0.002%. Although differences in the relative abundances were persistent when clustering at the 97% OTU level, fewer discrepancies were observed (Figure 3 and Supplementary Table 4). For example, nine out of ten phyla were detected in both protocols, and the two dominant phyla (Proteobacteria and Firmicutes) account for 79.1% and 75.15% for MiSeq and HiSeq protocols, respectively. Interestingly, in both ASV and OTU datasets, the most abundant phyla were more evenly represented across samples from the HiSeq protocol as opposed to the MiSeq protocol (see “n” in Supplementary Table 4). However, ASV libraries derived from the HiSeq and MiSeq protocols also contained several unclassified bacterial ASVs that were resolved when clustering at the 97% OTU level (see “Unclassified Bacteria” in Figure 3). Further investigation into the make-up of these unclassified reads using NCBI blast (Altschul et al., 1990) found a few close hits to eukaryotes, yet the majority remained unidentified; comparing reads against the more prokaryote-focused RDP database (Cole et al., 2014) did not better resolve unclassified bacteria (< 0.1% of hits passed a 97% identity threshold). To ensure these putative eukaryotes that passed our automated quality control measures did not affect the results of this study, we also manually removed them and re-ran statistical tests (Supplementary Table 5) and re-plotted relative abundance (Supplementary Figure 4). This additional quality control measure did not change the results of this study. Regardless, this suggests there may be challenges in the taxonomic assignment of ASVs from short read data and requires further attention.

### Standard Normalizations Do Not Overcome Protocol Induced Variability in Microbiome Diversity

To examine whether standard normalization methods used in the field could overcome the differences between protocols, the datasets were manipulated by either removing rare taxa or grouping at higher taxonomic classifications, including Family and Phylum level. Truncating the microbial communities by removing rare taxa did not eliminate the beta diversity differences between the two protocols (Supplementary Table 6). Removing rare taxa reduced the communities to less than 20 taxa (at ASV or OTU level), representing less than 1% of the total and suggesting that these bacterial communities are predominantly composed of low abundance taxa. Datasets of both species and taxonomic assignments (ASVs and OTUs) maintained the previously seen significant differences between protocols for all dissimilarity metrics aside from Weighted UniFrac for *M. aequituberculata* for both 0.5% and 1% rare taxa cut-offs (Supplementary Table 6). To reduce the effects of minor differences in closely related bacterial taxa, we also ran PERMANOVAs and homogeneity of variance tests on communities at both the Family and Phylum classification level. Significant differences were found again between protocols, however, this varied according to both host species and taxonomic level (Supplementary Table 7). *Porites lobata* showed significant differences between protocols even at the Phylum level, whereas *M. aequituberculata* communities were significantly different between protocols at the Family level, but only the two dissimilarity metrics utilizing presence/absence data (binary Jaccard and Unweighted UniFrac) showed significant differences at the Phylum level.

### Differential Abundance Analysis Is Not Protocol Agnostic

**FIGURE 4:**
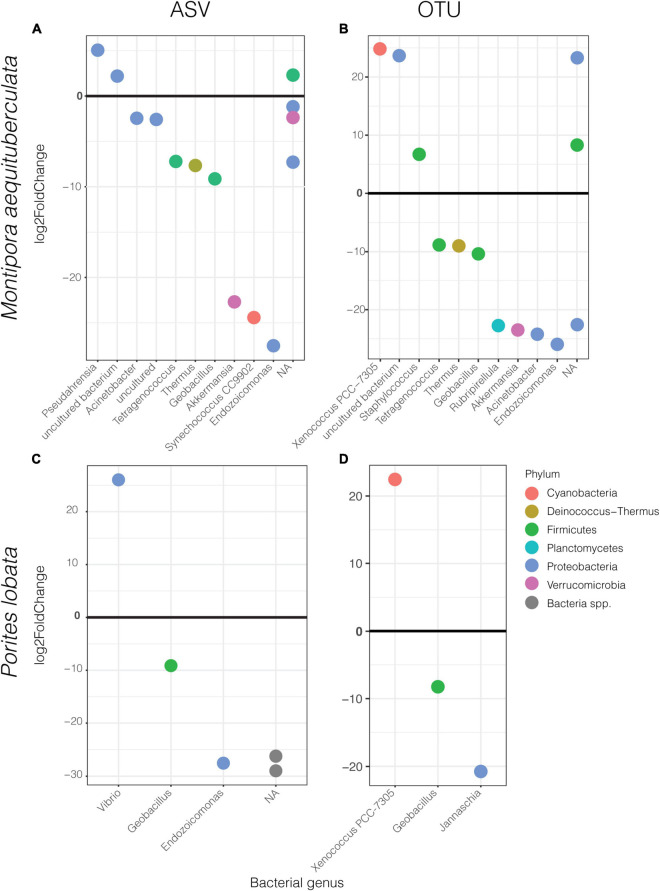
Significantly different ASVs (left) and OTUs (right) between protocols labeled by bacterial genus and colored by phylum for *M. aequituberculata* **(A,B)** and *P. lobata* **(C,D)**. Positive log2fold change refers to those significantly enriched in MiSeq protocol samples and negative log2fold change are those significantly enriched in HiSeq protocol samples. NA refers to bacteria unclassified at genus level.

Differential abundance analyses showed that only a few specific ASVs were significantly enriched in one protocol or the other (Figure 4 and Supplementary Table 8). The most enriched taxa belong to the dominant phyla, Proteobacteria and Firmicutes, with the magnitude of enrichments ranging between an approximately 7- and 29-fold change. While there was variation in differentially abundant taxa between protocols according to species and clustering method, some taxa were consistently different. For instance, HiSeq protocol libraries for both species had consistently higher abundances of *Geobacillus* sp., and lower abundances of Xenococcus PCC-7305 using the OTU dataset (Figures 4B,D). The magnitude of these enrichments was also consistent between coral species (Supplementary Table 8). Importantly, differential enrichment between the protocols was observed in two taxa identified as crucial players of coral health and resilience, *Endozoicomonas* and *Vibrio* spp. *Endozoicomonas* exhibited significantly higher abundances in data derived from the HiSeq protocol in both species and datasets (ASV vs. OTU), except in *P. lobata* using the OTU dataset (Figures 4A–C). *Porites lobata* showed significantly higher abundances of a *Vibrio* ASV when sequences were prepared with the MiSeq protocol (Figure 4C), but this difference was not maintained in the OTU dataset (Figure 4D). A closer look at all *Vibrio* and *Endozoicomonas* ASVs found in the sequencing output from HiSeq, MiSeq, or both protocols revealed no significant differences in mean GC content or melting temperatures (Supplementary Table 9).

## Discussion

In Scleractinian corals, 16S rRNA gene profiling remains a common and cost-effective tool for quantifying diversity of bacteria and some archaea in holobionts (Hernandez-Agreda et al., 2019). With the increase in sequencing of coral hosts by large collaborative groups such as EMP, and subsequent public sharing of sequencing data, it has become a common goal to examine widespread patterns through meta-analyses that combine datasets from multiple laboratories, making direct comparability a necessity.

In this small-scale comparative analysis of technical replicates, we found the greatest differences between the HiSeq and MiSeq protocols in the beta diversity and dispersion measures of coral microbiomes. Specifically, beta diversity and dispersion metrics were inconsistent between protocols, host species, and dissimilarity metrics, with differences in protocol explaining between 4 and 28% of the microbiome variability. Certain taxa were also significantly enriched in only one of the protocols, including those with known ecological importance. For example, *Vibrio* spp. and *Endozoicomonas* spp. ASVs were significantly enriched in MiSeq and HiSeq protocols, respectively. These two taxa have been identified as important in the health and maintenance of coral homeostasis and are often used to make statements about the health of the coral host (Bourne et al., 2008, 2016): *Vibrio* spp. have been implicated in disease (Ben-Haim et al., 2003) but remain common partners in healthy corals, while *Endozoicomonas* spp. are hypothesized to benefit to coral health via synthesis of dimethylsulfoniopropionate (DMSP) (Tandon et al., 2020), carbohydrate cycling and protein provisioning (Neave et al., 2017a), and may be considered a potential symbiont (Neave et al., 2017b). The differential abundances of these two taxa between protocols are particularly troubling for coral-specific studies and further indicate that care must be taken when comparing coral microbiome datasets resulting from even highly similar protocols.

Alpha diversity metrics on ASV data (both abundance-based and phylogenetic) were consistent between protocols in both coral species. Alpha diversity using OTU data were comparable between protocols with all metrics for *M. aequituberculata*, but significant differences were present with Shannon Diversity and Faith’s PD indices for *P. lobata*, suggesting some variability in alpha diversity in regard to both relative abundances and phylogenetic makeup when sequences are grouped with 97% similarity only. Nonetheless, our results suggest that comparisons of some alpha diversity metrics between protocols may be more reliable than comparisons of community composition. The results found here should be benchmarked in other systems and tested more broadly across species to determine the extent to which small differences in protocol might bias the perceived composition of host-associated or environmental microbiome sequencing.

Regardless of rarefaction, removal of low abundance reads, or comparisons of the resulting data at higher taxonomic levels, the bacterial community composition and relative abundances of taxa maintained differences between the two protocols but in ways that were inconsistent across host species and analytical metric. This can result, for instance, from one set of samples containing taxa that may never be present if they are prepared with a different protocol or sequenced on a different platform, likely due to differences in sequencing depth, where the deeper sequencing of the HiSeq platform can provide a greater opportunity to identify rare taxa (Caporaso et al., 2012), thus shifting the overall community composition. We note that while we used a relatively high threshold for removal of rare taxa in the present study, it may be useful to use a lower threshold (e.g., removing taxa with below 0.1 or 0.01% relative abundance) depending on dataset. However, differences were also apparent between OTU and ASV datasets, suggesting that how we characterize bacterial species and/or strains, and at what taxonomic level we choose to analyze these data, may result in unintended biases. We found no evidence of differences in secondary structure of two differentially abundant taxa (*Vibrio* and *Endozoicomonas*) that could have resulted from differential denaturation of sequences in the two platforms due to differences in platform chemistry (Nakamura et al., 2011). Specifically, there was no indication of high GC content in these sequences, which has previously been found to affect read numbers from Illumina sequencing runs due to intermittent halting of polymerase during amplification (Lyubetsky et al., 2006; Price et al., 2017). While this was not an exhaustive dive into the effects of platform chemistry on sequencing outcome, it suggests that differential abundances of specific taxa are unlikely to be caused by the presence of differential secondary structures. However, further research is necessary to rule this out completely.

The samples used in this study were not initially intended to test differences between protocols or sequencing platforms, but rather provided an opportunity to examine an overlapping set of technical replicates that arose from a larger study comprised of similarly prepared and differentially sequenced samples. Thus, we cannot clearly identify the specific mechanism(s) involved in driving the found community differences. Biases in these complex microbial communities could be a result of (1) differences in sequencing depth that are not overcome by rarefaction or other *in silico* normalizations, (2) library denaturation and/or sequencing platform chemistry, (3) differences in reagents and/or batches of reagents, such as the type of Hi Fidelity Taq used in PCR or other extraction, PCR or library preparation reagents, and potentially even (4) user and/or facility bias (Rausch et al., 2016; Parker et al., 2018). Regardless, the results shown here reveal not only the necessity to design a targeted study to examine procedural and mechanistic differences in sequencing protocols, but also the responsibility of researchers to proceed with extreme caution when combining and interpreting datasets that are generated from subtly and seemingly innocuously different methodologies.

## Conclusion

The present study found limitations in our ability to compare coral microbiome ‘technical’ replicates that were generated in almost identical fashions but then sequenced on different platforms. Despite attempts to rectify these issues with some commonly used normalization methods, we still found significant differences in some alpha diversity metrics and in most beta diversity metrics between the two protocols. These inconsistencies make it difficult to identify a “cure-all” adjustment for comparability between even highly similar protocols and, instead, differences among protocols and sequencing platforms are more likely to be specific to the microbiome host and specific set of microbiomes found in each dataset. Studies that aim to compare beta diversity may find more confidence in their results if overlapping technical replicates for each dataset and host species are run to ensure correct adjustments are used for these specific datasets. Based on these results, we urge caution in the statistical comparison and interpretation of 16S rRNA gene datasets that combine data resulting from different protocols and sequencing platforms. While we continue to encourage meta-analyses to discover of cosmopolitan patterns in microbiome dynamics, we advise researchers to be cognizant that even minor variations in the protocol can significantly affect microbiome composition, and those running longitudinal studies be rigorous in the consistency of their methods through time.

## Data Availability Statement

The raw data from the Illumina MiSeq samples are publicly available from McDevitt-Irwin et al. (2019) at Harvard DataVerse: https://doi.org/10.7910/DVN/3QZTT1. The raw data from the Illumina HiSeq samples are available on the NCBI Sequence Read Archive (SRA) under the BioProject accession PRJNA687031. All bioinformatics and statistical codes are freely accessible via github at github.com/hannaheps/comparison. The original contributions presented in the study are included in the article/Supplementary Material, further inquiries can be directed to the corresponding author.

## Author Contributions

JB was responsible for the original sample collection and access to sequencing data used in this manuscript. HE and RVT developed the research question. HE performed bioinformatic and statistical analyses with technical support from SS and AH-A and wrote the initial draft of the manuscript and received editorial support from all co-authors. All co-authors contributed to the interpretation of results.

## Conflict of Interest

The authors declare that the research was conducted in the absence of any commercial or financial relationships that could be construed as a potential conflict of interest.

## Publisher’s Note

All claims expressed in this article are solely those of the authors and do not necessarily represent those of their affiliated organizations, or those of the publisher, the editors and the reviewers. Any product that may be evaluated in this article, or claim that may be made by its manufacturer, is not guaranteed or endorsed by the publisher.

## Footnotes

1
https://www.hmpdacc.org/hmp

2
https://earthmicrobiome.org/
