# Supplementary material for: Inconsistent Patterns of Microbial Diversity and Composition Between Highly Similar Sequencing Protocols: A Case Study With Reef-Building Corals
Source: Front Microbiol. 2021 Nov 25;12:740932. doi: 10.3389/fmicb.2021.740932 (PMC8656265; doi:10.3389/fmicb.2021.740932)
Supplement: Supplementary file 1 [file Data_Sheet_1.docx]

Supplementary Tables

Table S1: Mean (± SE), median and range of denoised, non-chimeric, non-eukaryotic and decontaminated sequence reads assigned to each sample by protocol and host coral species used in downstream analyses.

| Host Species | Dataset | Protocol | Sample n | Mean (± SE) | Median | Range  (min – max) |
| --- | --- | --- | --- | --- | --- | --- |
| *Montipora aequituberculata* | Amplicon Sequence Variants (ASVs) | HiSeq | 11 | 48,899.45 ± 16,395.11 | 21,829 | 1,140 – 144,255 |
|  |  | MiSeq | 11 | 11,644.82 ± 4,180.10 | 5,711 | 1,287 – 42,896 |
|  | Operational Taxonomic Units (OTUs) | HiSeq | 11 | 40,145.73 ± 13,788.23 | 18,415 | 1,072 – 130,520 |
|  |  | MiSeq | 11 | 8,946.27 ± 3,378.30 | 5,191 | 1,042 – 39,233 |
| *Porites lobata* | Amplicon Sequence Variants (ASVs) | HiSeq | 13 | 47,863.31 ± 21,686.10 | 14,498 | 1,005 – 28,4210 |
|  |  | MiSeq | 13 | 12,021.77 ± 4,426.238 | 6,560 | 1,543 – 61,732 |
|  | Operational Taxonomic Units (OTUs) | HiSeq | 11 | 25,100.64 ± 10,474.48 | 6,588 | 998 – 87,837 |
|  |  | MiSeq | 11 | 12,479.73 ± 4,598.69 | 6,515 | 1,556 – 55,247 |

Table S2: Linear model results for alpha diversity metrics between the HiSeq and MiSeq protocols. Bold *p* values with asterisks (*) refer to significant results (α = 0.05).

| Host Species | Dataset | Diversity Index | Transformation  (Shapiro-Wilk statistic, p value) | DF | t statistic | *p* value |
| --- | --- | --- | --- | --- | --- | --- |
| *Montipora aequituberculata* | Amplicon Sequence Variants (ASVs) | Species Richness | Log  (W = 0.94, *p* = 0.23) | 10 | 0.592 | 0.567 |
|  |  | Shannon Diversity | NA  (W = 0.98, *p* = 0.93) | 10 | 0.876 | 0.401 |
|  |  | Faith’s PD | Inverse log  (W = 0.93, *p* = 0.15) | 10 | 0.068 | 0.947 |
|  | Operational Taxonomic Units (OTUs) | Species Richness | Square root  (W = 0.99, *p* = 0.98) | 10 | 0.985 | 0.975 |
|  |  | Shannon Diversity | NA  (W = 0.99, *p* = 0.99) | 10 | 0.988 | 0.993 |
|  |  | Faith’s PD | Log  (W = 0.92, *p* = 0.10) | 10 | -0.691 | 0.506 |
| *Porites lobata* | Amplicon Sequence Variants (ASVs) | Species Richness | Log  (W = 0.95, *p* = 0.23) | 12 | 1.177 | 0.262 |
|  |  | Shannon Diversity | Log  (W = 0.93, *p* = 0.10) | 12 | 1.002 | 0.336 |
|  |  | Faith’s PD | Log  (W = 0.97, *p* = 0.53) | 12 | 1.583 | 0.140 |
|  | Operational Taxonomic Units (OTUs) | Species Richness | Log  (W = 0.94, *p* = 0.21) | 10 | 2.044 | 0.068 |
|  |  | Shannon Diversity | NA  (W = 0.95, *p* = 0.39) | 10 | 2.267 | **0.047*** |
|  |  | Faith’s PD | Log  (W = 0.93, *p* = 0.11) | 10 | 2.485 | **0.032*** |

Table S3: Permutational analyses of variance (PERMANOVA) and homogeneity of variance (PERMDISP) results for A) unrarefied and B) rarefied community data between protocol of *P. lobata* and *M. aequituberculata* for four dissimilarity metrics: binary Jaccard, Bray Curtis, unweighted and weighted Unifrac. Bold *p* values with asterisks (*) refer to significant results (α = 0.05).

**A.**

| Host Species | Dataset | Dissimilarity Metric | Test | DF | F Statistic | R^2^ | *p* value |
| --- | --- | --- | --- | --- | --- | --- | --- |
| *Montipora aequituberculata* | Amplicon Sequence Variants (ASVs) | Jaccard | PERMANOVA | 1 | 1.171 | 0.06 | **0.002*** |
|  |  |  | PERMDISP | 1 | 1.29 |  | 0.731 |
|  |  | Bray Curtis | PERMANOVA | 1 | 1.140 | 0.05 | **0.007*** |
|  |  |  | PERMDISP | 1 | 2e-04 |  | 0.986 |
|  |  | Unweighted Unifrac | PERMANOVA | 1 | 0.214 | 0.10 | **0.006*** |
|  |  |  | PERMDISP | 1 | 10.386 |  | **0.003*** |
|  |  | Weighted Unifrac | PERMANOVA | 1 | 0.853 | 0.04 | 0.293 |
|  |  |  | PERMDISP | 1 | 0.192 |  | 0.685 |
|  | Operational Taxonomic Units (OTUs) | Jaccard | PERMANOVA | 1 | 1.236 | 0.06 | **0.001*** |
|  |  |  | PERMDISP | 1 | 1.502 |  | 0.238 |
|  |  | Bray Curtis | PERMANOVA | 1 | 1.179 | 0.06 | **0.002*** |
|  |  |  | PERMDISP | 1 | 1.021 |  | 0.884 |
|  |  | Unweighted Unifrac | PERMANOVA | 1 | 1.095 | 0.05 | **0.019*** |
|  |  |  | PERMDISP | 1 | 0.363 |  | 0.547 |
|  |  | Weighted Unifrac | PERMANOVA | 1 | 1.428 | 0.07 | 0.098 |
|  |  |  | PERMDISP | 1 | 8.424 |  | **0.002*** |
| *Porites lobata* | Amplicon Sequence Variants (ASVs) | Jaccard | PERMANOVA | 1 | 1.444 | 0.06 | **0.001*** |
|  |  |  | PERMDISP | 1 | 0.833 |  | 0.371 |
|  |  | Bray Curtis | PERMANOVA | 1 | 3.408 | 0.12 | **0.004*** |
|  |  |  | PERMDISP | 1 | 7.585 |  | **0.012*** |
|  |  | Unweighted Unifrac | PERMANOVA | 1 | 1.475 | 0.06 | **0.001*** |
|  |  |  | PERMDISP | 1 | 0.216 |  | 0.654 |
|  |  | Weighted Unifrac | PERMANOVA | 1 | 0.719 | 0.03 | **0.002*** |
|  |  |  | PERMDISP | 1 | 0.282 |  | 0.604 |
|  | Operational Taxonomic Units (OTUs) | Jaccard | PERMANOVA | 1 | 1.487 | 0.07 | **0.001*** |
|  |  |  | PERMDISP | 1 | 0.080 |  | 0.778 |
|  |  | Bray Curtis | PERMANOVA | 1 | 2.068 | 0.09 | **0.001*** |
|  |  |  | PERMDISP | 1 | 6.973 |  | **0.008*** |
|  |  | Unweighted Unifrac | PERMANOVA | 1 | 1.392 | 0.07 | **0.005*** |
|  |  |  | PERMDISP | 1 | 17.77 |  | **0.001*** |
|  |  | Weighted Unifrac | PERMANOVA | 1 | 1.279 | 0.06 | **0.003*** |
|  |  |  | PERMDISP | 1 | 0.399 |  | 0.546 |

**B.**

| Host Species | Dataset | Dissimilarity Metric | Test | DF | F Statistic | R^2^ | *p* value |
| --- | --- | --- | --- | --- | --- | --- | --- |
| *Montipora aequituberculata* | Amplicon Sequence Variants (ASVs) | Jaccard | PERMANOVA | 1 | 1.158 | 0.05 | **0.002*** |
|  |  |  | PERMDISP | 1 | 0.664 |  | 0.429 |
|  |  | Bray Curtis | PERMANOVA | 1 | 1.150 | 0.05 | **0.007*** |
|  |  |  | PERMDISP | 1 | 0.005 |  | 0.939 |
|  |  | Unweighted Unifrac | PERMANOVA | 1 | 1.165 | 0.06 | 0.08 |
|  |  |  | PERMDISP | 1 | 1.212 |  | 0.29 |
|  |  | Weighted Unifrac | PERMANOVA | 1 | 0.497 | 0.02 | 0.453 |
|  |  |  | PERMDISP | 1 | 0.378 |  | 0.564 |
|  | Operational Taxonomic Units (OTUs) | Jaccard | PERMANOVA | 1 | 1.258 | 0.06 | **0.001*** |
|  |  |  | PERMDISP | 1 | 2.480 |  | 0.128 |
|  |  | Bray Curtis | PERMANOVA | 1 | 1.167 | 0.06 | **0.002*** |
|  |  |  | PERMDISP | 1 | 0.124 |  | 0.729 |
|  |  | Unweighted Unifrac | PERMANOVA | 1 | 1.289 | 0.06 | **0.004*** |
|  |  |  | PERMDISP | 1 | 0.057 |  | 0.833 |
|  |  | Weighted Unifrac | PERMANOVA | 1 | 1.428 | 0.07 | 0.105 |
|  |  |  | PERMDISP | 1 | 8.424 |  | **0.011*** |
| *Porites lobata* | Amplicon Sequence Variants (ASVs) | Jaccard | PERMANOVA | 1 | 1.483 | 0.06 | **0.001*** |
|  |  |  | PERMDISP | 1 | 1.819 |  | 0.188 |
|  |  | Bray Curtis | PERMANOVA | 1 | 3.438 | 0.13 | **0.001*** |
|  |  |  | PERMDISP | 1 | 7.587 |  | **0.008*** |
|  |  | Unweighted Unifrac | PERMANOVA | 1 | 1.37 | 0.05 | 0.096 |
|  |  |  | PERMDISP | 1 | 0.028 |  | 0.864 |
|  |  | Weighted Unifrac | PERMANOVA | 1 | 0.994 | 0.04 | **0.002*** |
|  |  |  | PERMDISP | 1 | 0.765 |  | 0.417 |
|  | Operational Taxonomic Units (OTUs) | Jaccard | PERMANOVA | 1 | 1.471 | 0.07 | **0.001*** |
|  |  |  | PERMDISP | 1 | 0.561 |  | 0.467 |
|  |  | Bray Curtis | PERMANOVA | 1 | 2.044 | 0.09 | **0.001*** |
|  |  |  | PERMDISP | 1 | 6.942 |  | **0.011*** |
|  |  | Unweighted Unifrac | PERMANOVA | 1 | 1.602 | 0.07 | **0.003*** |
|  |  |  | PERMDISP | 1 | 15.02 |  | **0.004*** |
|  |  | Weighted Unifrac | PERMANOVA | 1 | 1.426 | 0.07 | **0.004*** |
|  |  |  | PERMDISP | 1 | 0.298 |  | 0.597 |

Table S4: Mean relative abundances (%), standard deviation and standard error of the top 10 most abundant phyla for each dataset (ASV and OTU) and each protocol (MiSeq and HiSeq), ranked from most abundant to least abundant. “n” refers to the number of samples that contained the given phylum. Total n for each dataset is included in the table below.

| Dataset | Protocol | Rank | Phylum | n | Mean | sd | se |
| --- | --- | --- | --- | --- | --- | --- | --- |
| Amplicon Sequence Variants (ASVs)  Total n = 24 | MiSeq | **1** | Proteobacteria | 24 | 67.48 | 28.21 | 5.76 |
|  |  | **2** | Firmicutes | 20 | 7.23 | 9.77 | 2.18 |
|  |  | **3** | Cyanobacteria | 21 | 5.65 | 7.30 | 1.59 |
|  |  | **4** | Planctomycetes | 16 | 4.73 | 4.58 | 1.14 |
|  |  | **5** | Bacteroidetes | 22 | 4.57 | 7.04 | 1.50 |
|  |  | **6** | Euryarchaeota | 4 | 4.45 | 8.71 | 4.35 |
|  |  | **7** | Verrucomicrobia | 15 | 3.70 | 3.23 | 0.84 |
|  |  | **8** | Epsilonbacteraeota | 6 | 2.80 | 6.38 | 2.61 |
|  |  | **9** | Actinobacteria | 17 | 2.57 | 3.01 | 0.73 |
|  |  | **10** | Chlamydiae | 11 | 1.41 | 2.32 | 0.70 |
|  | HiSeq | **1** | Proteobacteria | 24 | 45.07 | 28.95 | 5.91 |
|  |  | **2** | Firmicutes | 24 | 9.60 | 14.27 | 2.91 |
|  |  | **3** | Bacteroidetes | 24 | 6.068 | 10.13 | 2.07 |
|  |  | **4** | Verrucomicrobia | 21 | 4.71 | 7.58 | 1.65 |
|  |  | **5** | Cyanobacteria | 24 | 3.23 | 4.06 | 0.83 |
|  |  | **6** | Actinobacteria | 18 | 2.77 | 4.68 | 0.98 |
|  |  | **7** | Planctomycetes | 18 | 2.34 | 3.15 | 0.72 |
|  |  | **8** | Dienococcus-Thermus | 18 | 2.03 | 3.17 | 0.75 |
|  |  | **9** | Fusobacteria | 13 | 1.34 | 2.68 | 0.74 |
|  |  | **10** | Tenericutes | 12 | 1.22 | 2.18 | 0.63 |
| Operational Taxonomic Units (OTUs)  Total n = 22 | MiSeq | **1** | Proteobacteria | 22 | 69.69 | 25.25 | 5.38 |
|  |  | **2** | Firmicutes | 18 | 9.41 | 11.29 | 2.66 |
|  |  | **3** | Euryarchaeota | 4 | 8.46 | 16.69 | 8.35 |
|  |  | **4** | Cyanobacteria | 18 | 7.21 | 8.37 | 1.97 |
|  |  | **5** | Verrucomicrobia | 14 | 5.34 | 5.04 | 1.35 |
|  |  | **6** | Planctomycetes | 15 | 4.76 | 4.62 | 1.19 |
|  |  | **7** | Bacteroidetes | 18 | 4.42 | 6.24 | 1.47 |
|  |  | **8** | Epsilonbacteraeota | 6 | 3.39 | 7.84 | 3.20 |
|  |  | **9** | Actinobacteria | 16 | 3.15 | 3.32 | 0.83 |
|  |  | **10** | Fusobacteria | 10 | 1.23 | 2.37 | 0.75 |
|  | HiSeq | **1** | Proteobacteria | 22 | 61.58 | 26.31 | 5.61 |
|  |  | **2** | Firmicutes | 22 | 13.57 | 15.29 | 3.26 |
|  |  | **3** | Bacteroidetes | 22 | 5.49 | 8.86 | 1.89 |
|  |  | **4** | Verrucomicrobia | 21 | 5.24 | 7.76 | 1.69 |
|  |  | **5** | Cyanobacteria | 22 | 3.88 | 4.58 | 0.98 |
|  |  | **6** | Actinobacteria | 21 | 3.63 | 5.12 | 1.12 |
|  |  | **7** | Planctomycetes | 16 | 3.09 | 2.86 | 0.72 |
|  |  | **8** | Deinococcus-Thermus | 18 | 2.84 | 4.39 | 1.04 |
|  |  | **9** | Fusobacteria | 12 | 1.70 | 2.92 | 0.84 |
|  |  | **10** | Epsilonbacteraeota | 7 | 1.50 | 2.08 | 0.78 |

Table S5: Permutational analyses of variance (PERMANOVA) and homogeneity of variance (PERMDISP) results for A) unrarefied and B) rarefied community data with unassigned eukaryotic reads removed between protocol of *P. lobata* and *M. aequituberculata* for four dissimilarity metrics: binary Jaccard, Bray Curtis, unweighted and weighted Unifrac. These data include only ASVs, and bold *p* values with asterisks (*) refer to significant results (α = 0.05).

**A.**

| Host Species | Dataset | Dissimilarity Metric | Test | DF | F Statistic | R^2^ | *p* value |
| --- | --- | --- | --- | --- | --- | --- | --- |
| *Montipora aequituberculata* | Amplicon Sequence Variants (ASVs) | Jaccard | PERMANOVA | 1 | 1.69 | 0.06 | **0.002*** |
|  |  |  | PERMDISP | 1 | 0.11 |  | 0.749 |
|  |  | Bray Curtis | PERMANOVA | 1 | 1.141 | 0.05 | **0.009*** |
|  |  |  | PERMDISP | 1 | 0.002 |  | 0.968 |
|  |  | Unweighted Unifrac | PERMANOVA | 1 | 2.145 | 0.10 | **0.012*** |
|  |  |  | PERMDISP | 1 | 10.74 |  | **0.007*** |
|  |  | Weighted Unifrac | PERMANOVA | 1 | 0.836 | 0.04 | 0.291 |
|  |  |  | PERMDISP | 1 | 0.288 |  | 0.605 |
| *Porites lobata* | Amplicon Sequence Variants (ASVs) | Jaccard | PERMANOVA | 1 | 1.406 | 006 | **0.001*** |
|  |  |  | PERMDISP | 1 | 0.869 |  | 0.349 |
|  |  | Bray Curtis | PERMANOVA | 1 | 2.461 | 0.09 | **0.001*** |
|  |  |  | PERMDISP | 1 | 8.073 |  | **0.01*** |
|  |  | Unweighted Unifrac | PERMANOVA | 1 | 1.552 | 0.06 | **0.002*** |
|  |  |  | PERMDISP | 1 | 0.648 |  | 0.421 |
|  |  | Weighted Unifrac | PERMANOVA | 1 | 0.808 | 0.03 | **0.012*** |
|  |  |  | PERMDISP | 1 | 1.173 |  | 0.339 |

**B.**

| Host Species | Dataset | Dissimilarity Metric | Test | DF | F Statistic | R^2^ | *p* value |
| --- | --- | --- | --- | --- | --- | --- | --- |
| *Montipora aequituberculata* | Amplicon Sequence Variants (ASVs) | Jaccard | PERMANOVA | 1 | 1.155 | 0.05 | **0.002*** |
|  |  |  | PERMDISP | 1 | 0.681 |  | 0.468 |
|  |  | Bray Curtis | PERMANOVA | 1 | 1.150 | 0.05 | **0.007*** |
|  |  |  | PERMDISP | 1 | 0.016 |  | 0.890 |
|  |  | Unweighted Unifrac | PERMANOVA | 1 | 1.174 | 0.06 | 0.082 |
|  |  |  | PERMDISP | 1 | 1.586 |  | 0.205 |
|  |  | Weighted Unifrac | PERMANOVA | 1 | 0.514 | 0.03 | 0.503 |
|  |  |  | PERMDISP | 1 | 0.361 |  | 0.567 |
| *Porites lobata* | Amplicon Sequence Variants (ASVs) | Jaccard | PERMANOVA | 1 | 1.417 | 0.06 | **0.001*** |
|  |  |  | PERMDISP | 1 | 1.827 |  | 0.174 |
|  |  | Bray Curtis | PERMANOVA | 1 | 2.430 | 0.09 | **0.001*** |
|  |  |  | PERMDISP | 1 | 8.288 |  | **0.008*** |
|  |  | Unweighted Unifrac | PERMANOVA | 1 | 1.358 | 0.05 | 0.092 |
|  |  |  | PERMDISP | 1 | 1e -04 |  | 0.994 |
|  |  | Weighted Unifrac | PERMANOVA | 1 | 0.962 | 0.04 | **0.011*** |
|  |  |  | PERMDISP | 1 | 2.615 |  | 0.140 |

Table S6: Permutational analyses of variance (PERMANOVA) and homogeneity of variance (PERMDISP) results for A) unrarefied and B) rarefied community data between protocol from ASV and OTU datasets discarding rare taxa, defined as those taxa that had relative abundances below either 0.5% or 1% (rare taxa cut-off). Bold *p* values with asterisks (*) refer to significant results (α = 0.05).

**A.**

| Rare  Taxa Cut-off | Host Species | Dataset | Dissimilarity Metric | Test | DF | F Statistic | R^2^ | *p* value |
| --- | --- | --- | --- | --- | --- | --- | --- | --- |
| 0.5% | *Montipora aequituberculata* | Amplicon Sequence Variants (ASVs) | Jaccard | PERMANOVA | 1 | 43.14 | 0.14 | **0.005*** |
|  |  |  |  | PERMDISP | 1 | 1.29 |  | 0.285 |
|  |  |  | Bray Curtis | PERMANOVA | 1 | 1.40 | 0.07 | **0.009*** |
|  |  |  |  | PERMDISP | 1 | 1.87 |  | 0.181 |
|  |  |  | Unweighted Unifrac | PERMANOVA | 1 | 2.74 | 0.12 | **0.003*** |
|  |  |  |  | PERMDISP |  | 0.02 |  | 0.88 |
|  |  |  | Weighted Unifrac | PERMANOVA | 1 | 0.97 | 0.05 | 0.113 |
|  |  |  |  | PERMDISP | 1 | 2.94 |  | 0.09 |
|  |  | Operational Taxonomic Units (OTUs) | Jaccard | PERMANOVA | 1 | 3.97 | 0.17 | **0.002*** |
|  |  |  |  | PERMDISP | 1 | 1.22 |  | 0.289 |
|  |  |  | Bray Curtis | PERMANOVA | 1 | 1.39 | 0.06 | **0.01*** |
|  |  |  |  | PERMDISP | 1 | 0.89 |  | 0.364 |
|  |  |  | Unweighted Unifrac | PERMANOVA | 1 | 3.13 | 0.14 | **0.005*** |
|  |  |  |  | PERMDISP | 1 | 1.73 |  | 0.193 |
|  |  |  | Weighted Unifrac | PERMANOVA | 1 | 1.12 | 0.05 | 0.054 |
|  |  |  |  | PERMDISP | 1 | 0.86 |  | 0.366* |
|  | *Porites lobata* | Amplicon Sequence Variants (ASVs) | Jaccard | PERMANOVA | 1 | 4.36 | 0.15 | **0.001*** |
|  |  |  |  | PERMDISP | 1 | 0.08 |  | 0.787 |
|  |  |  | Bray Curtis | PERMANOVA | 1 | 4.68 | 0.16 | **0.001*** |
|  |  |  |  | PERMDISP | 1 | 7.63 |  | **0.01*** |
|  |  |  | Unweighted Unifrac | PERMANOVA | 1 | 7.08 | 0.23 | **0.002*** |
|  |  |  |  | PERMDISP | 1 | 4.82 |  | 0.052 |
|  |  |  | Weighted Unifrac | PERMANOVA | 1 | 6.11 | 0.20 | **0.001*** |
|  |  |  |  | PERMDISP | 1 | 4.82 |  | **0.032*** |
|  |  | Operational Taxonomic Units (OTUs) | Jaccard | PERMANOVA | 1 | 4.39 | 0.17 | **0.001*** |
|  |  |  |  | PERMDISP | 1 | 0.43 |  | 0.495 |
|  |  |  | Bray Curtis | PERMANOVA | 1 | 2.68 | 0.11 | **0.003*** |
|  |  |  |  | PERMDISP | 1 | 8.73 |  | **0.008*** |
|  |  |  | Unweighted Unifrac | PERMANOVA | 1 | 4.18 | 0.17 | **0.001*** |
|  |  |  |  | PERMDISP | 1 | 26.60 |  | **0.001*** |
|  |  |  | Weighted Unifrac | PERMANOVA | 1 | 1.31 | 0.10 | **0.001*** |
|  |  |  |  | PERMDISP | 1 | 5.05 |  | **0.023*** |
| 1% | *Montipora aequituberculata* | Amplicon Sequence Variants (ASVs) | Jaccard | PERMANOVA | 1 | 3.09 | 0.13 | **0.007*** |
|  |  |  |  | PERMDISP |  | 2.59 |  | 0.119 |
|  |  |  | Bray Curtis | PERMANOVA | 1 | 1.30 | 0.06 | **0.026*** |
|  |  |  |  | PERMDISP | 1 | 0.01 |  | 0.763 |
|  |  |  | Unweighted Unifrac | PERMANOVA | 1 | 3.43 | 0.15 | **0.003*** |
|  |  |  |  | PERMDISP | 1 | 0.19 |  | 0.66 |
|  |  |  | Weighted Unifrac | PERMANOVA | 1 | 1.11 | 0.05 | 0.104 |
|  |  |  |  | PERMDISP | 1 | 2.05 |  | 0.161 |
|  |  | Operational Taxonomic Units (OTUs) | Jaccard | PERMANOVA | 1 | 2.62 | 0.12 | **0.007*** |
|  |  |  |  | PERMDISP | 1 | 2.75 |  | 0.13 |
|  |  |  | Bray Curtis | PERMANOVA | 1 | 1.43 | 0.07 | **0.012*** |
|  |  |  |  | PERMDISP | 1 | 0.62 |  | 0.47 |
|  |  |  | Unweighted Unifrac | PERMANOVA | 1 | 3.68 | 0.16 | **0.005*** |
|  |  |  |  | PERMDISP | 1 | 3.26 |  | 0.078 |
|  |  |  | Weighted Unifrac | PERMANOVA | 1 | 1.51 | 0.07 | **0.034*** |
|  |  |  |  | PERMDISP | 1 | 0.22 |  | 0.621 |
|  | Porites lobata | Amplicon Sequence Variants (ASVs) | Jaccard | PERMANOVA | 1 | 4.89 | 0.17 | **0.001*** |
|  |  |  |  | PERMDISP | 1 | 0.36 |  | 0.533 |
|  |  |  | Bray Curtis | PERMANOVA | 1 | 3.97 | 0.14 | **0.001*** |
|  |  |  |  | PERMDISP | 1 | 7.04 |  | **0.019*** |
|  |  |  | Unweighted Unifrac | PERMANOVA | 1 | 9.51 | 0.28 | **0.001*** |
|  |  |  |  | PERMDISP | 1 | 4.23 |  | **0.047*** |
|  |  |  | Weighted Unifrac | PERMANOVA | 1 | 6.22 | 0.21 | **0.002*** |
|  |  |  |  | PERMDISP | 1 | 4.80 |  | **0.049*** |
|  |  | Operational Taxonomic Units (OTUs) | Jaccard | PERMANOVA | 1 | 3.73 | 0.15 | **0.002*** |
|  |  |  |  | PERMDISP | 1 | 1.26 |  | 0.27 |
|  |  |  | Bray Curtis | PERMANOVA | 1 | 2.11 | 0.10 | **0.008*** |
|  |  |  |  | PERMDISP | 1 | 5.42 |  | **0.035*** |
|  |  |  | Unweighted Unifrac | PERMANOVA | 1 | 5.55 | 0.22 | **0.002*** |
|  |  |  |  | PERMDISP | 1 | 24.46 |  | **0.001*** |
|  |  |  | Weighted Unifrac | PERMANOVA | 1 | 1.99 | 0.09 | **0.001*** |
|  |  |  |  | PERMDISP | 1 | 3.76 |  | 0.069 |

**B.**

| Rare  Taxa Cut-off | Host Species | Dataset | Dissimilarity Metric | Test | DF | F Statistic | R^2^ | *p* value |
| --- | --- | --- | --- | --- | --- | --- | --- | --- |
| 0.5% | *Montipora aequituberculata* | Amplicon Sequence Variants (ASVs) | Jaccard | PERMANOVA | 1 | 3.83 | 0.16 | **0.005*** |
|  |  |  |  | PERMDISP | 1 | 1.37 |  | 0.216 |
|  |  |  | Bray Curtis | PERMANOVA | 1 | 1.40 | 0.07 | **0.009*** |
|  |  |  |  | PERMDISP | 1 | 1.87 |  | 0.181 |
|  |  |  | Unweighted Unifrac | PERMANOVA | 1 | 2.90 | 0.13 | **0.009*** |
|  |  |  |  | PERMDISP |  | 2.07 |  | 0.157 |
|  |  |  | Weighted Unifrac | PERMANOVA | 1 | 1.19 | 0.06 | 0.056 |
|  |  |  |  | PERMDISP | 1 | 1.15 |  | 0.321 |
|  |  | Operational Taxonomic Units (OTUs) | Jaccard | PERMANOVA | 1 | 3.828 | 0.16 | **0.005*** |
|  |  |  |  | PERMDISP | 1 | 1.37 |  | 0.216 |
|  |  |  | Bray Curtis | PERMANOVA | 1 | 4.94 | 0.17 | **0.001*** |
|  |  |  |  | PERMDISP | 1 | 8.61 |  | 0.178 |
|  |  |  | Unweighted Unifrac | PERMANOVA | 1 | 2.90 | 0.13 | **0.009*** |
|  |  |  |  | PERMDISP | 1 | 2.07 |  | 0.157 |
|  |  |  | Weighted Unifrac | PERMANOVA | 1 | 1.19 | 0.06 | 0.056 |
|  |  |  |  | PERMDISP | 1 | 1.15 |  | 0.321 |
|  | *Porites lobata* | Amplicon Sequence Variants (ASVs) | Jaccard | PERMANOVA | 1 | 4.09 | 0.15 | **0.006*** |
|  |  |  |  | PERMDISP | 1 | 0.35 |  | 0.559 |
|  |  |  | Bray Curtis | PERMANOVA | 1 | 4.94 | 0.17 | **0.001*** |
|  |  |  |  | PERMDISP | 1 | 8.61 |  | **0.009*** |
|  |  |  | Unweighted Unifrac | PERMANOVA | 1 | 8.86 | 0.27 | **0.002*** |
|  |  |  |  | PERMDISP | 1 | 1.54 |  | 0.211 |
|  |  |  | Weighted Unifrac | PERMANOVA | 1 | 6.30 | 0.21 | **0.001*** |
|  |  |  |  | PERMDISP | 1 | 5.23 |  | **0.029*** |
|  |  | Operational Taxonomic Units (OTUs) | Jaccard | PERMANOVA | 1 | 3.71 | 0.16 | **0.002*** |
|  |  |  |  | PERMDISP | 1 | 1.01 |  | 0.314 |
|  |  |  | Bray Curtis | PERMANOVA | 1 | 2.20 | 0.10 | **0.003*** |
|  |  |  |  | PERMDISP | 1 | 6.66 |  | **0.020*** |
|  |  |  | Unweighted Unifrac | PERMANOVA | 1 | 4.41 | 0.18 | **0.001*** |
|  |  |  |  | PERMDISP | 1 | 13.15 |  | **0.003*** |
|  |  |  | Weighted Unifrac | PERMANOVA | 1 | 1.85 | 0.08 | **0.002*** |
|  |  |  |  | PERMDISP | 1 | 3.65 |  | **0.076*** |
| 1% | *Montipora aequituberculata* | Amplicon Sequence Variants (ASVs) | Jaccard | PERMANOVA | 1 | 3.03 | 0.13 | **0.004*** |
|  |  |  |  | PERMDISP | 1 | 1.36 |  | 0.255 |
|  |  |  | Bray Curtis | PERMANOVA | 1 | 1.44 | 0.07 | **0.014*** |
|  |  |  |  | PERMDISP | 1 | 1.98 |  | 0.182 |
|  |  |  | Unweighted Unifrac | PERMANOVA | 1 | 3.17 | 0.14 | **0.003*** |
|  |  |  |  | PERMDISP | 1 | 0.19 |  | 0.66 |
|  |  |  | Weighted Unifrac | PERMANOVA | 1 | 1.12 | 0.05 | 0.098 |
|  |  |  |  | PERMDISP | 1 | 2.71 |  | 0.109 |
|  |  | Operational Taxonomic Units (OTUs) | Jaccard | PERMANOVA | 1 | 2.44 | 0.11 | **0.01*** |
|  |  |  |  | PERMDISP | 1 | 1.78 |  | 0.187 |
|  |  |  | Bray Curtis | PERMANOVA | 1 | 1.38 | 0.06 | **0.029*** |
|  |  |  |  | PERMDISP | 1 | 1.14 |  | 0.284 |
|  |  |  | Unweighted Unifrac | PERMANOVA | 1 | 3.67 | 0.15 | **0.002*** |
|  |  |  |  | PERMDISP | 1 | 1.97 |  | 0.196 |
|  |  |  | Weighted Unifrac | PERMANOVA | 1 | 1.52 | 0.07 | **0.023*** |
|  |  |  |  | PERMDISP | 1 | 0.66 |  | 0.441 |
|  | Porites lobata | Amplicon Sequence Variants (ASVs) | Jaccard | PERMANOVA | 1 | 4.09 | 0.15 | **0.002*** |
|  |  |  |  | PERMDISP | 1 | 0.35 |  | 0.526 |
|  |  |  | Bray Curtis | PERMANOVA | 1 | 4.94 | 0.17 | **0.001*** |
|  |  |  |  | PERMDISP | 1 | 8.61 |  | **0.005*** |
|  |  |  | Unweighted Unifrac | PERMANOVA | 1 | 8.86 | 0.27 | **0.001*** |
|  |  |  |  | PERMDISP | 1 | 1.54 |  | 0.215 |
|  |  |  | Weighted Unifrac | PERMANOVA | 1 | 6.30 | 0.21 | **0.001*** |
|  |  |  |  | PERMDISP | 1 | 5.28 |  | **0.032*** |
|  |  | Operational Taxonomic Units (OTUs) | Jaccard | PERMANOVA | 1 | 4.08 | 0.17 | **0.003*** |
|  |  |  |  | PERMDISP | 1 | 0.28 |  | 0.624 |
|  |  |  | Bray Curtis | PERMANOVA | 1 | 2.00 | 0.09 | **0.01*** |
|  |  |  |  | PERMDISP | 1 | 4.51 |  | **0.042*** |
|  |  |  | Unweighted Unifrac | PERMANOVA | 1 | 5.62 | 0.22 | **0.003*** |
|  |  |  |  | PERMDISP | 1 | 25.06 |  | **0.001*** |
|  |  |  | Weighted Unifrac | PERMANOVA | 1 | 1.87 | 0.09 | **0.003*** |
|  |  |  |  | PERMDISP | 1 | 3.36 |  | 0.073 |

Table S7: Permutational analyses of variance (PERMANOVA) and homogeneity of variance (PERMDISP) results for A) unrarefied and B) rarefied community data between protocol from ASV and OTU datasets using Family and Phylum taxonomic levels. Bold *p* values with asterisks (*) refer to significant results (α = 0.05).

**A.**

| Taxonomic Rank | Host Species | Dataset | Dissimilarity Metric | Test | DF | F Statistic | R^2^ | *p* value |
| --- | --- | --- | --- | --- | --- | --- | --- | --- |
| Family | *Montipora aequituberculata* | Amplicon Sequence Variants (ASVs) | Jaccard | PERMANOVA | 1 | 1.513 | 0.07 | **0.002*** |
|  |  |  |  | PERMDISP | 1 | 0.626 |  | 0.456 |
|  |  |  | Bray Curtis | PERMANOVA | 1 | 1.242 | 0.06 | **0.037*** |
|  |  |  |  | PERMDISP | 1 | 0.066 |  | 0.781 |
|  |  |  | Unweighted Unifrac | PERMANOVA | 1 | 1.516 | 0.07 | **0.003*** |
|  |  |  |  | PERMDISP |  | 0.188 |  | 0.751 |
|  |  |  | Weighted Unifrac | PERMANOVA | 1 | 1.079 | 0.05 | 0.162 |
|  |  |  |  | PERMDISP | 1 | 0.713 |  | 0.38 |
|  |  | Operational Taxonomic Units (OTUs) | Jaccard | PERMANOVA | 1 | 1.585 | 0.07 | **0.004*** |
|  |  |  |  | PERMDISP | 1 | 1.827 |  | 0.206 |
|  |  |  | Bray Curtis | PERMANOVA | 1 | 1.182 | 0.06 | 0.063 |
|  |  |  |  | PERMDISP | 1 | 0.020 |  | 0.892 |
|  |  |  | Unweighted Unifrac | PERMANOVA | 1 | 1.913 | 0.09 | **0.002*** |
|  |  |  |  | PERMDISP | 1 | 0.544 |  | 0.525 |
|  |  |  | Weighted Unifrac | PERMANOVA | 1 | 1.067 | 0.05 | 0.125 |
|  |  |  |  | PERMDISP | 1 | 0.004 |  | 0.954 |
|  | *Porites lobata* | Amplicon Sequence Variants (ASVs) | Jaccard | PERMANOVA | 1 | 1.775 | 0.07 | **0.003*** |
|  |  |  |  | PERMDISP | 1 | 15.932 |  | **0.001*** |
|  |  |  | Bray Curtis | PERMANOVA | 1 | 3.262 | 0.12 | **0.001*** |
|  |  |  |  | PERMDISP | 1 | 9.406 |  | **0.002*** |
|  |  |  | Unweighted Unifrac | PERMANOVA | 1 | 2.235 | 0.09 | **0.004*** |
|  |  |  |  | PERMDISP | 1 | 5.409 |  | **0.035*** |
|  |  |  | Weighted Unifrac | PERMANOVA | 1 | 2.353 | 0.09 | **0.004*** |
|  |  |  |  | PERMDISP | 1 | 4.705 |  | **0.045*** |
|  |  | Operational Taxonomic Units (OTUs) | Jaccard | PERMANOVA | 1 | 2.023 | 0.09 | **0.001*** |
|  |  |  |  | PERMDISP | 1 | 20.98 |  | **0.001*** |
|  |  |  | Bray Curtis | PERMANOVA | 1 | 2.690 | 0.12 | **0.001*** |
|  |  |  |  | PERMDISP | 1 | 4.719 |  | **0.041*** |
|  |  |  | Unweighted Unifrac | PERMANOVA | 1 | 2.097 | 0.09 | **0.002*** |
|  |  |  |  | PERMDISP | 1 | 21.66 |  | **0.001*** |
|  |  |  | Weighted Unifrac | PERMANOVA | 1 | 2.042 | 0.09 | **0.006*** |
|  |  |  |  | PERMDISP | 1 | 2.746 |  | 0.107 |
| Phylum | *Montipora aequituberculata* | Amplicon Sequence Variants (ASVs) | Jaccard | PERMANOVA | 1 | 2.367 | 0.11 | **0.021*** |
|  |  |  |  | PERMDISP | 1 | 0.392 |  | 0.859 |
|  |  |  | Bray Curtis | PERMANOVA | 1 | 0.923 | 0.04 | 0.295 |
|  |  |  |  | PERMDISP | 1 | 1.677 |  | 0.226 |
|  |  |  | Unweighted Unifrac | PERMANOVA | 1 | 2.863 | 0.13 | **0.024*** |
|  |  |  |  | PERMDISP | 1 | 0.05 |  | 0.848 |
|  |  |  | Weighted Unifrac | PERMANOVA | 1 | 0.846 | 0.04 | 0.422 |
|  |  |  |  | PERMDISP | 1 | 2.858 |  | 0.121 |
|  |  | Operational Taxonomic Units (OTUs) | Jaccard | PERMANOVA | 1 | 3.333 | 0.14 | **0.004*** |
|  |  |  |  | PERMDISP | 1 | 1.114 |  | 0.332 |
|  |  |  | Bray Curtis | PERMANOVA | 1 | 0.789 | 0.04 | 0.384 |
|  |  |  |  | PERMDISP | 1 | 2.803 |  | 0.116 |
|  |  |  | Unweighted Unifrac | PERMANOVA | 1 | 3.851 | 0.16 | **0.012*** |
|  |  |  |  | PERMDISP | 1 | 1.558 |  | 0.227 |
|  |  |  | Weighted Unifrac | PERMANOVA | 1 | 1.042 | 0.05 | 0.313 |
|  |  |  |  | PERMDISP | 1 | 1.463 |  | 0.265 |
|  | *Porites lobata* | Amplicon Sequence Variants (ASVs) | Jaccard | PERMANOVA | 1 | 2.356 | 0.09 | **0.01*** |
|  |  |  |  | PERMDISP | 1 | 22.39 |  | **0.002*** |
|  |  |  | Bray Curtis | PERMANOVA | 1 | 4.887 | 0.17 | **0.001*** |
|  |  |  |  | PERMDISP | 1 | 19.573 |  | **0.001*** |
|  |  |  | Unweighted Unifrac | PERMANOVA | 1 | 4.8871 | 0.17 | **0.001*** |
|  |  |  |  | PERMDISP | 1 | 19.573 |  | **0.001*** |
|  |  |  | Weighted Unifrac | PERMANOVA | 1 | 2.525 | 0.10 | **0.012*** |
|  |  |  |  | PERMDISP | 1 | 1.953 |  | 0.192 |
|  |  | Operational Taxonomic Units (OTUs) | Jaccard | PERMANOVA | 1 | 3.325 | 0.14 | **0.002*** |
|  |  |  |  | PERMDISP | 1 | 34.19 |  | **0.001*** |
|  |  |  | Bray Curtis | PERMANOVA | 1 | 1.231 | 0.06 | **0.02*** |
|  |  |  |  | PERMDISP | 1 | 0.143 |  | 0.605 |
|  |  |  | Unweighted Unifrac | PERMANOVA | 1 | 2.791 | 0.12 | **0.001*** |
|  |  |  |  | PERMDISP | 1 | 26.39 |  | **0.001*** |
|  |  |  | Weighted Unifrac | PERMANOVA | 1 | 1.559 | 0.07 | **0.006*** |
|  |  |  |  | PERMDISP | 1 | 0.078 |  | 0.758 |

**B.**

| Taxonomic Rank | Host Species | Dataset | Dissimilarity Metric | Test | DF | F Statistic | R^2^ | *p* value |
| --- | --- | --- | --- | --- | --- | --- | --- | --- |
| Family | *Montipora aequituberculata* | Amplicon Sequence Variants (ASVs) | Jaccard | PERMANOVA | 1 | 1.43 | 0.07 | **0.007*** |
|  |  |  |  | PERMDISP | 1 | 0.266 |  | 0.597 |
|  |  |  | Bray Curtis | PERMANOVA | 1 | 1.26 | 0.06 | **0.028*** |
|  |  |  |  | PERMDISP | 1 | 0.031 |  | 0.864 |
|  |  |  | Unweighted Unifrac | PERMANOVA | 1 | 1.41 | 0.07 | **0.009*** |
|  |  |  |  | PERMDISP |  | 0.277 |  | 0.616 |
|  |  |  | Weighted Unifrac | PERMANOVA | 1 | 1.128 | 0.05 | 0.088 |
|  |  |  |  | PERMDISP | 1 | 0.671 |  | 0.428 |
|  |  | Operational Taxonomic Units (OTUs) | Jaccard | PERMANOVA | 1 | 1.546 | 0.07 | **0.002*** |
|  |  |  |  | PERMDISP | 1 | 1.827 |  | 0.206 |
|  |  |  | Bray Curtis | PERMANOVA | 1 | 1.239 | 0.06 | **0.043*** |
|  |  |  |  | PERMDISP | 1 | 0.001 |  | 0.968 |
|  |  |  | Unweighted Unifrac | PERMANOVA | 1 | 1.801 | 0.09 | **0.002*** |
|  |  |  |  | PERMDISP | 1 | 0.654 |  | 0.418 |
|  |  |  | Weighted Unifrac | PERMANOVA | 1 | 1.102 | 0.05 | 0.085 |
|  |  |  |  | PERMDISP | 1 | 0.001 |  | 0.981 |
|  | *Porites lobata* | Amplicon Sequence Variants (ASVs) | Jaccard | PERMANOVA | 1 | 2.00 | 0.08 | **0.001*** |
|  |  |  |  | PERMDISP | 1 | 6.85 |  | **0.017*** |
|  |  |  | Bray Curtis | PERMANOVA | 1 | 3.181 | 0.12 | **0.001*** |
|  |  |  |  | PERMDISP | 1 | 9.820 |  | **0.003*** |
|  |  |  | Unweighted Unifrac | PERMANOVA | 1 | 1.787 | 0.07 | **0.005*** |
|  |  |  |  | PERMDISP | 1 | 6.138 |  | **0.025*** |
|  |  |  | Weighted Unifrac | PERMANOVA | 1 | 2.89 | 0.11 | **0.001*** |
|  |  |  |  | PERMDISP | 1 | 4.345 |  | 0.054 |
|  |  | Operational Taxonomic Units (OTUs) | Jaccard | PERMANOVA | 1 | 1.953 | 0.09 | **0.002*** |
|  |  |  |  | PERMDISP | 1 | 10.66 |  | **0.005*** |
|  |  |  | Bray Curtis | PERMANOVA | 1 | 2.619 | 0.12 | **0.002*** |
|  |  |  |  | PERMDISP | 1 | 4.716 |  | **0.038*** |
|  |  |  | Unweighted Unifrac | PERMANOVA | 1 | 2.211 | 0.10 | **0.004*** |
|  |  |  |  | PERMDISP | 1 | 15.59 |  | **0.001*** |
|  |  |  | Weighted Unifrac | PERMANOVA | 1 | 1.970 | 0.09 | **0.006*** |
|  |  |  |  | PERMDISP | 1 | 2.724 |  | 0.106 |
| Phylum | *Montipora aequituberculata* | Amplicon Sequence Variants (ASVs) | Jaccard | PERMANOVA | 1 | 2.085 | 0.09 | **0.026*** |
|  |  |  |  | PERMDISP | 1 | 0.059 |  | 0.827 |
|  |  |  | Bray Curtis | PERMANOVA | 1 | 0.886 | 0.04 | 0.322 |
|  |  |  |  | PERMDISP | 1 | 1.541 |  | 0.244 |
|  |  |  | Unweighted Unifrac | PERMANOVA | 1 | 2.757 | 0.12 | **0.028*** |
|  |  |  |  | PERMDISP | 1 | 1.022 |  | 0.355 |
|  |  |  | Weighted Unifrac | PERMANOVA | 1 | 0.859 | 0.04 | 0.441 |
|  |  |  |  | PERMDISP | 1 | 4.041 |  | 0.066 |
|  |  | Operational Taxonomic Units (OTUs) | Jaccard | PERMANOVA | 1 | 3.235 | 0.14 | **0.005*** |
|  |  |  |  | PERMDISP | 1 | 1.636 |  | 0.469 |
|  |  |  | Bray Curtis | PERMANOVA | 1 | 0.710 | 0.34 | 0.444 |
|  |  |  |  | PERMDISP | 1 | 2.192 |  | 0.160 |
|  |  |  | Unweighted Unifrac | PERMANOVA | 1 | 3.159 | 0.14 | **0.013*** |
|  |  |  |  | PERMDISP | 1 | 0.706 |  | 0.441 |
|  |  |  | Weighted Unifrac | PERMANOVA | 1 | 0.921 | 0.05 | 0.305 |
|  |  |  |  | PERMDISP | 1 | 1.463 |  | 0.385 |
|  | *Porites lobata* | Amplicon Sequence Variants (ASVs) | Jaccard | PERMANOVA | 1 | 3.007 | 0.11 | **0.009*** |
|  |  |  |  | PERMDISP | 1 | 12.95 |  | **0.003*** |
|  |  |  | Bray Curtis | PERMANOVA | 1 | 4.765 | 0.17 | **0.004*** |
|  |  |  |  | PERMDISP | 1 | 18.327 |  | **0.001*** |
|  |  |  | Unweighted Unifrac | PERMANOVA | 1 | 4.765 | 0.17 | **0.003*** |
|  |  |  |  | PERMDISP | 1 | 7.566 |  | **0.006*** |
|  |  |  | Weighted Unifrac | PERMANOVA | 1 | 2.444 | 0.09 | **0.005*** |
|  |  |  |  | PERMDISP | 1 | 1.594 |  | 0.229 |
|  |  | Operational Taxonomic Units (OTUs) | Jaccard | PERMANOVA | 1 | 3.290 | 0.14 | **0.002*** |
|  |  |  |  | PERMDISP | 1 | 31.534 |  | **0.001*** |
|  |  |  | Bray Curtis | PERMANOVA | 1 | 1.04 | 0.05 | **0.028*** |
|  |  |  |  | PERMDISP | 1 | 0.090 |  | 0.696 |
|  |  |  | Unweighted Unifrac | PERMANOVA | 1 | 3.045 | 0.13 | **0.004*** |
|  |  |  |  | PERMDISP | 1 | 32.45 |  | **0.001*** |
|  |  |  | Weighted Unifrac | PERMANOVA | 1 | 1.329 | 0.06 | **0.012*** |
|  |  |  |  | PERMDISP | 1 | 0.062 |  | 0.802 |

Table S8: Significantly different taxa resulting from DeSeq2 [1] analysis. Positive log2fold change refers to those significantly enriched in MiSeq protocol samples and negative log2fold change are those significantly enriched in HiSeq protocol samples. Taxonomic assignments for ASVs and OTUs are done to the highest resolution available using the SILVA database. *Vibrio* and *Endozoicomonas* taxa are identified in bold.

| Host Species | Dataset | Taxa | Log2Fold Change | *p* value  (adj) |
| --- | --- | --- | --- | --- |
| *Montipora aequituberculata* | Amplicon Sequence Variants (ASVs) | K:Bacteria; P:Deinococcus; C:Deinococci; O:Thermales; F:Thermaceae; G:*Thermus*; S:*Thermus thermophilus* | -7.66 | 0.006 |
|  |  | K:Bacteria; P:Proteobacteria | -7.29 | 0.01 |
|  |  | K:Bacteria; P:Cyanobacteria; C:Oxyphotobacteria; O:Synechococcales; F:Cyanobiaceae; G:*Synechococcus* CC9902 | -24.42 | 1.67e^-14^ |
|  |  | K:Bacteria; P:Firmicutes; C:Bacilli; O:Lactobacillales; F:Enterococcaceae; G:*Tetragenococcus*; S: *Tetragenococcus halophilus* subsp. *haliophilus* | -7.21 | 7.36e^-05^ |
|  |  | K:Bacteria; P:Firmicutes; C:Bacilli; O:Bacillales; F:Bacillaceae; G:*Geobacillus* | -9.13 | 2.06e^-11^ |
|  |  | K:Bacteria; P:Verrucomicrobia; C:Verrucomicrobiae; O:Verrumicrobiales; F:Akkermansiaceae; G:*Akkermansia* | -22.7 | 1.76e^-15^ |
|  |  | **K:Bacteria; P:Proteobacteria; C:Gammaproteobacteria; O:Oceanospirillales; F:Endozoicomonadaceae; G:*Endozoicomonas*** | **-27.53** | **2.82e^-27^** |
|  | Operational Taxonomic Units (OTUs) | K:Bacteria; P:Cyanobacteria; C:Oxyphotobacteria; O:Nostocales; F:Xenococcaceae; G:*Xenococcus* PCC-7305 | 24.62 | 5.19e^-17^ |
|  |  | K:Bacteria; P:Proteobacteria; C:Deltaproteobacteria; O:Oligoflexales; F:Oligoflexaceae | 23.73 | 3.93e^-14^ |
|  |  | K:Bacteria; P: Proteobacteria; C:Alphaproteobacteria | 23.34 | 7.57e^-14^ |
|  |  | K:Bacteria; P:Firmicutes | 8.23 | 0.046 |
|  |  | K:Bacteria; P:Firmicutes; C:Bacilli; O:Bacillales; F: Staphylococcaceae; G:*Staphylococcus* | 6.70 | 0.018 |
|  |  | **K:Bacteria; P:Proteobacteria; C:Gammaproteobacteria; O:Oceanospirillales; F:Endozoicomondaceae; G:*Endozoicomonas*** | **-26.00** | **6.23e^-23^** |
|  |  | K:Bacteria; P:Proteobacteria; C:Gammaproteobacteria; O:Pseudomonadales; F:Moraxellaceae; G:*Acinetobacter* | -24.26 | 1.19e^-14^ |
|  |  | K:Bacteria; P:Verrucomicrobiae; O:Verrucomicrobiales; F:Akkermansiaceae; G:*Akkermansia* | -23.48 | 6.22e^-14^ |
|  |  | K:Bacteria; P:Proteobacteria; C:Gammaproteobacteria; O:Enterobacteriales; F:Enterobacteriaceae | -22.69 | 3.34e^-13^ |
|  |  | K:Bacteria; P:Plantomycetes; C:Planctomycetacia; O:Pirellulales; F:Pirellulaceae; G:*Rubripirellula* | -22.69 | 3.34e^-13^ |
|  |  | K:Bacteria; P:Firmicutes; C:Bacilli; O:Bacillales; F:Bacillaceae; G*:Geobacillus* | -10.35 | 4.42e^-05^ |
|  |  | K:Bacteria; P:Deinococcus-Thermus; C:Deinococci; O:Thermales; F:Thermaceae; G:*Thermus;* S:*Thermus thermophilus* | -9.01 | 0.009 |
|  |  | K:Bacteria; P:Firmicutes; C:Bacilli; O:Lactobacillales; F:Enterococcaceae; G:*Tetragenococcus*; S: *Tetragenococcus halophilus* subsp. *halophilus* | -8.83 | 0.001 |
| *Porites lobata* | Amplicon Sequence Variants (ASVs) | **K:Bacteria; P:Proteobacteria; C:Gammaproteobacteria; O:Vibrionales;**  **F: Vibrionaceae; G:*Vibrio*** | **26.02** | **3.84e^-17^** |
|  |  | K:Bacteria | -28.98 | 9.34e^-23^ |
|  |  | **K:Bacteria; P: Proteobacteria; C:Gammaproteobacteria; O:Oceanospirillales; F: Endozoicomonadaceae; G: *Endozoicomonas*** | **-27.53** | **2.82e^-27^** |
|  |  | K:Bacteria | -26.25 | 1.01e^-16^ |
|  |  | K:Bacteria | -26.23 | 1.01e^-16^ |
|  |  | K:Bacteria; P:Firmicutes; C:Bacilli; O:Bacillales; F:Bacillaceae; G: *Geobacillus* | -9.13 | 5.08e^-9^ |
|  | Operational Taxonomic Units (OTUs) | K:Bacteria; P:Cyanobacteria; C:Oxyphotobacteria; O:Nostocales; F:Xenococcaceae; G:*Xenococcus PCC-7305* | 22.45 | 2.38e^-11^ |
|  |  | K:Bacteria; P:Firmicutes; C:Bacilli; O:Bacillales; F:Bacillaceae | -8.27 | 2.27e^-02^ |
|  |  | K:Bacteria; P:Proteobacteria; C:Alphaproteobacteria; O:Rhodobacterales; F:Rhodobacteriaceae; G:*Jannaschia* | -20.75 | 9.10e^-10^ |

1. Love M, Ahlmann-Eltze C, Anders S, Huber W. DESeq2: Differential gene expression analysis based on the negative binomial distribution. Bioconductor version: Release (3.11); 2020. doi:10.18129/B9.bioc.DESeq2.

Table S9: Linear model results for GC content and melting temperatures of *Vibrio* ASVs and *Endozoicomonas* ASVs present in samples prepared using the HiSeq, MiSeq or both protocols. No significant results found at α = 0.05.

| Bacterial Taxon | Explanatory Variable | Transformation | df | F Statistic | *p* value |
| --- | --- | --- | --- | --- | --- |
| *Vibrio* spp. | GC Content | Log | 2 | 0.202 | 0.818 |
|  | Melting Temperature | Log | 2 | 0.220 | 0.803 |
| *Endozoicomonas* spp. | GC Content | Log | 2 | 2.8584 | 0.087 |
|  | Melting Temperature | Log | 2 | 2.8773 | 0.086 |
