# Supplementary figures and images for: Inconsistent Patterns of Microbial Diversity and Composition Between Highly Similar Sequencing Protocols: A Case Study With Reef-Building Corals
Source: Front Microbiol. 2021 Nov 25;12:740932. doi: 10.3389/fmicb.2021.740932 (PMC8656265; doi:10.3389/fmicb.2021.740932)

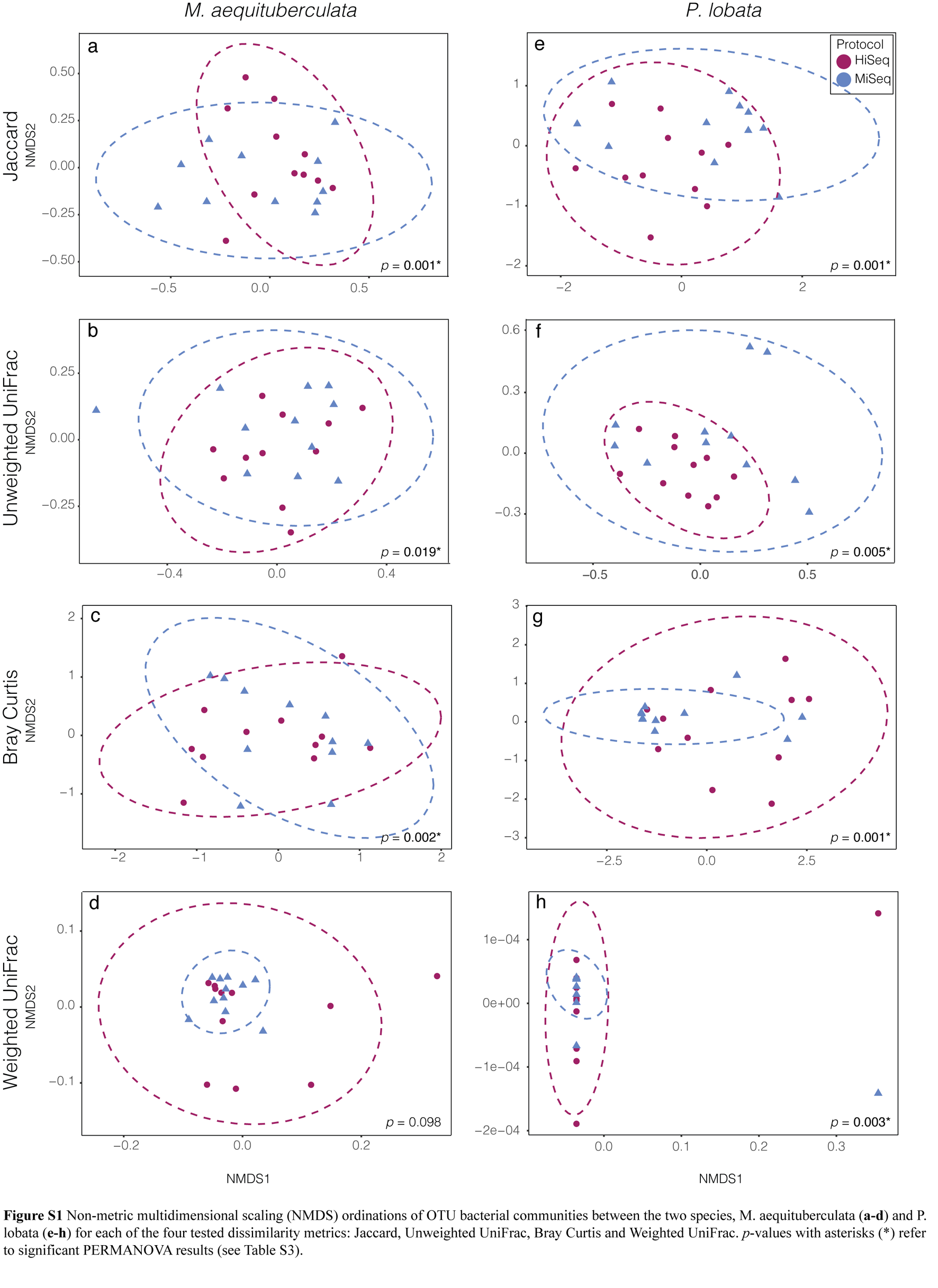

Supplement: Supplementary file 2 [file Image_1.TIF]

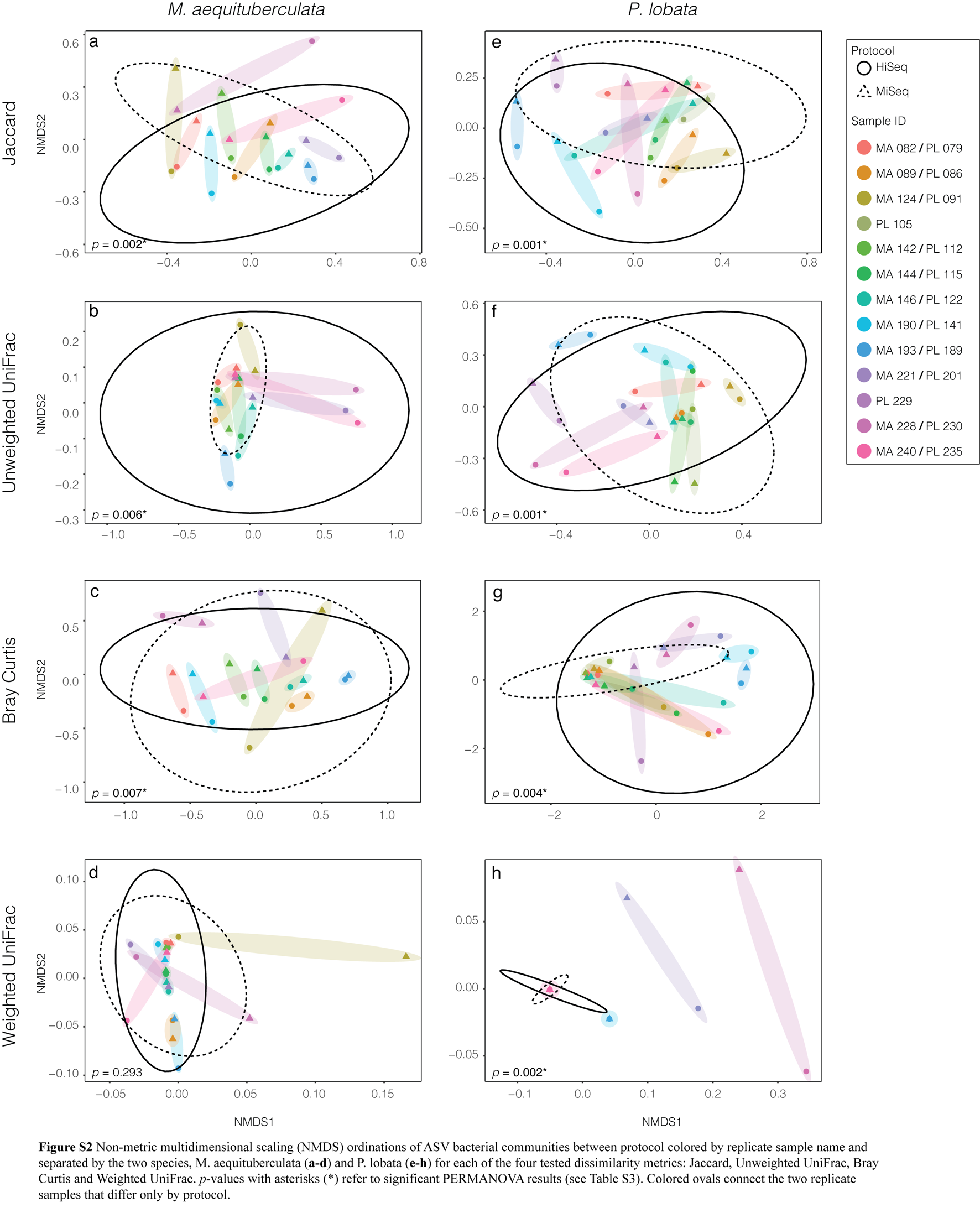

Supplement: Supplementary file 3 [file Image_2.TIF]

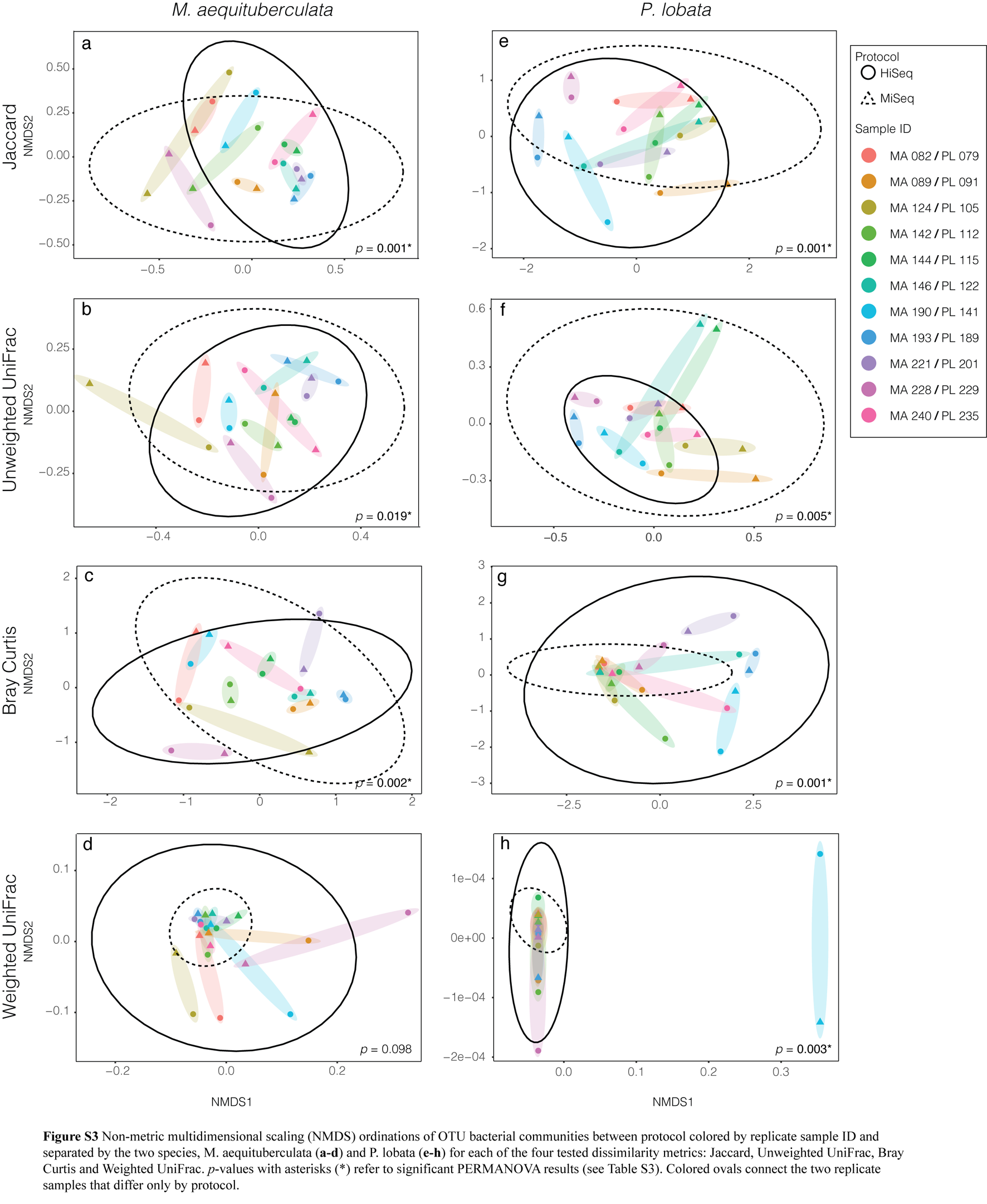

Supplement: Supplementary file 4 [file Image_3.TIF]

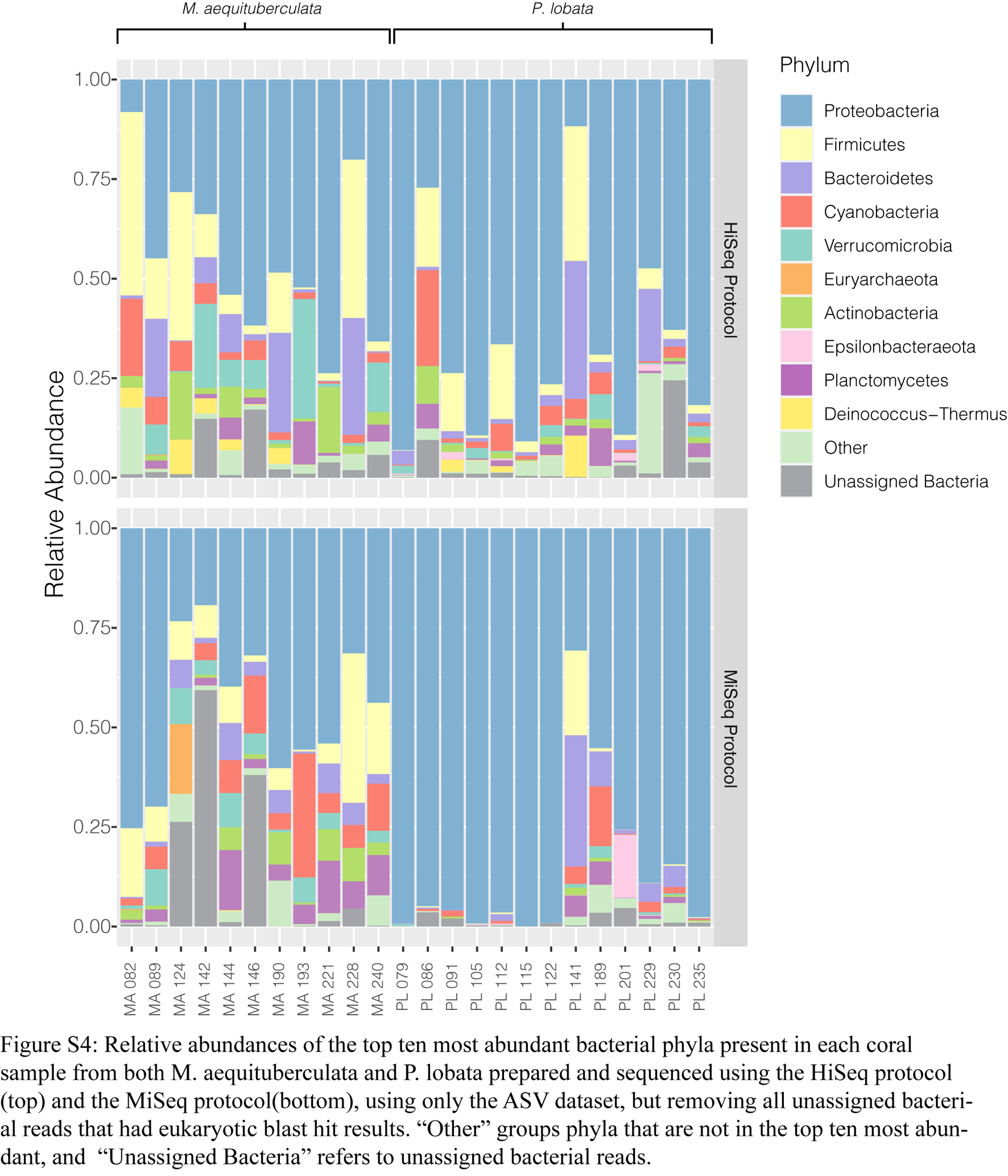

Supplement: Supplementary file 5 [file Image_4.TIF]
